# Purification of high-quality RNA from a small number of fluorescence activated cell sorted zebrafish cells for RNA sequencing purposes

**DOI:** 10.1186/s12864-019-5608-2

**Published:** 2019-03-20

**Authors:** Siebe Loontiens, Lisa Depestel, Suzanne Vanhauwaert, Givani Dewyn, Charlotte Gistelinck, Karen Verboom, Wouter Van Loocke, Filip Matthijssens, Andy Willaert, Jo Vandesompele, Frank Speleman, Kaat Durinck

**Affiliations:** 1 Department of Biomolecular Medicine & Center for Medical Genetics, Ghent University, 9000 Ghent, Belgium; 2 Cancer Research Institute Ghent (CRIG), 9000 Ghent, Belgium; 3 Department of Orthopedics and Sports Medicine, University of Washington, Seattle, WA 98195 USA

**Keywords:** RNA isolation, FACS sorting, Zebrafish, RNA sequencing

## Abstract

**Background:**

Transgenic zebrafish lines with the expression of a fluorescent reporter under the control of a cell-type specific promoter, enable transcriptome analysis of FACS sorted cell populations. RNA quality and yield are key determinant factors for accurate expression profiling. Limited cell number and FACS induced cellular stress make RNA isolation of sorted zebrafish cells a delicate process. We aimed to optimize a workflow to extract sufficient amounts of high-quality RNA from a limited number of FACS sorted cells from Tg*(fli1a:GFP)* zebrafish embryos, which can be used for accurate gene expression analysis.

**Results:**

We evaluated two suitable RNA isolation kits (the RNAqueous micro and the RNeasy plus micro kit) and determined that sorting cells directly into lysis buffer is a critical step for success. For low cell numbers, this ensures direct cell lysis, protects RNA from degradation and results in a higher RNA quality and yield. We showed that this works well up to 0.5× dilution of the lysis buffer with sorted cells. In our sort settings, this corresponded to 30,000 and 75,000 cells for the RNAqueous micro kit and RNeasy plus micro kit respectively. Sorting more cells dilutes the lysis buffer too much and requires the use of a collection buffer. We also demonstrated that an additional genomic DNA removal step after RNA isolation is required to completely clear the RNA from any contaminating genomic DNA. For cDNA synthesis and library preparation, we combined SmartSeq v4 full length cDNA library amplification, Nextera XT tagmentation and sample barcoding. Using this workflow, we were able to generate highly reproducible RNA sequencing results.

**Conclusions:**

The presented optimized workflow enables to generate high quality RNA and allows accurate transcriptome profiling of small populations of sorted zebrafish cells.

**Electronic supplementary material:**

The online version of this article (10.1186/s12864-019-5608-2) contains supplementary material, which is available to authorized users.

## Background

Over the recent years, it has become clear that transcriptional control relies on the orchestrated activity of transcription factor complexes, DNA methylation, chromatin modification dynamics as well as higher-order DNA looping [1]. Genetic lesions and epigenetic alterations have a major impact on rewiring of transcriptional networks during cancer development and importantly mining these perturbed transcriptomes allows to uncover novel therapeutic targets [2–4]. Technological advances in the field of massively parallel sequencing have greatly facilitated in-depth assessment of gene expression programs, with RNA sequencing presently serving as the gold standard for detailed and unbiased molecular characterization of both in vitro and in vivo model systems.

In recent years, zebrafish (*Danio rerio*) has become increasingly important as a model organism to study vertebrate development and cancer [5], as many cellular processes and developmental programs are evolutionary conserved. Furthermore, embryonic development in zebrafish is fast, with completion of embryogenesis within 5 days and adulthood reached in 3 months. The short generation time and large progeny combined with the ease to (non-invasively) study transparent embryos present obvious advantages compared to murine in vivo models [6, 7]. While zebrafish was initially primarily used for developmental studies, it is now also emerging as a relevant model to study human diseases [8–12]. The development of fluorescent reporter zebrafish lines, where a fluorescent marker is driven by a cell type-specific promoter, makes it feasible to perform fluorescence activated cell sorting (FACS), thereby circumventing the need of zebrafish specific antibodies for staining of specific cell populations. In this manner, straightforward enrichment of the cells of interest is feasible, enabling the definition of cell-specific transcriptomes that could otherwise be masked when ‘whole embryo’ derived RNA is analysed [13–16].

Despite the above-mentioned advantages, some considerations have to be made when using these zebrafish reporter lines for RNA-sequencing purposes. First, cells undergoing the process of sorting encounter stress and show reduced viability [17]. In dying cells, RNA decay is triggered and therefore inappropriate handling of the sample adds onto this RNA degradation process. The resulting transcriptome may therefore not be representative for the in vivo gene expression levels. In addition, RNA degradation does not occur at the same pace for every transcript and is defined by different aspects of the RNA transcript sequence (such as GC content, and length of the coding DNA sequence) [18–21]. This makes it very important to optimize a workflow that minimizes RNA degradation due to FACS induced cellular stress and cell death to obtain high quality RNA for expression analysis. Also, purified RNA is frequently contaminated with genomic DNA, possibly impacting on expression analysis results. Finally, sorting a large number of cells (> 1 million) is not always feasible when working with fluorescent reporter embryos. Depending on the promotor of choice, the fluorescent marker may only be expressed in a small number of cells and thereby result in a low RNA yield. Given the importance obtaining sufficient amounts of high quality RNA for expression studies, we optimized RNA extraction from FACS sorted cells from zebrafish embryos and did an in-depth quality assessment of the extracted RNA, the latter often omitted in performed experiments. We used the Tg(*fli1a:GFP*) zebrafish line to optimize our workflow. This zebrafish line expresses GFP in 10–15% of its cells, thereby requiring only a limited number of zebrafish embryos to obtain a sufficient number of cells for our experimental set-up. In this paper, we present a detailed workflow for gene expression analysis of a small number (5000–200,000) of fluorescence-based sorted zebrafish cells by means of RNA-sequencing, with a detailed description of the critical steps from embryo dissociation to the actual sequencing procedure.

## Results

### The RNAqueous micro kit and RNeasy plus micro kit enable high quality RNA purification from low numbers of FACS sorted EGFP positive zebrafish cells

In search for an appropriate method to isolate RNA from a low number (5000–200,000) of FACS sorted zebrafish cells, two RNA isolation kits were compared. The RNAqueous Micro Total RNA Isolation Kit from Ambion and the RNeasy Plus Micro Kit from Qiagen are specifically designed for purification of total RNA from a small amount of cells (< 200,000 cells). In both cases, only longer RNA fragments (> 200 nucleotides) are purified, although the workflow can be modified to isolate small RNAs such as microRNAs, 5,8S rRNA, 5S RNA, tRNA’s,… (Additional file 1: Figure S1). Ten replicates of 20,000 sorted cells from the *Tg*(*fli1a*:*EGFP*) transgenic zebrafish line were used as input for comparative RNA-isolation between the two kits. Each set of 20,000 cells was directly sorted into the lysis buffer of the RNA isolation kit (see further).

**Fig. 1:**
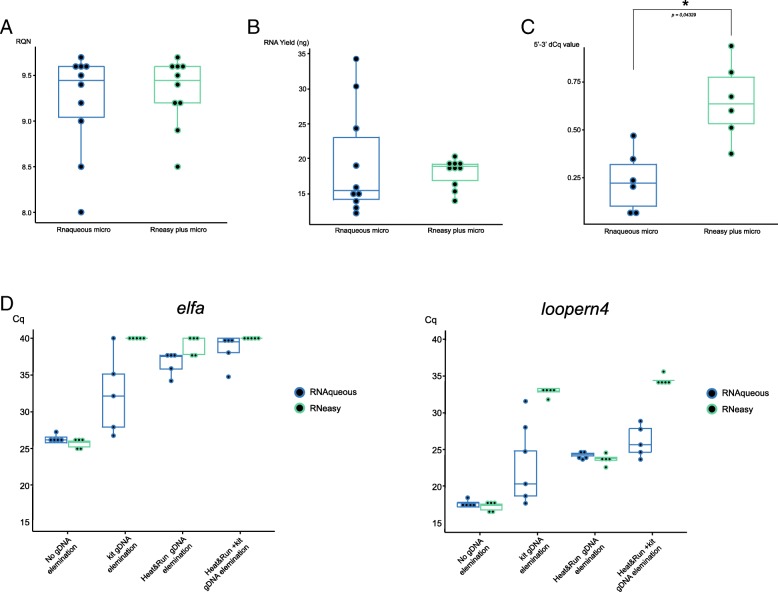
Comparative qualitative analysis of RNA purified with the Rnaqeuous and Rneasy micro kit. (**a**) Boxplots showing RQN values and (**b**) RNA yield of 10 RNA samples purified from 20,000 FACS sorted *fli1a:EGFP* zebrafish cells with the RNAqueous micro (*left*) and the RNeasy plus micro (*right*) kit. Red dots represent RQN values of individual samples. (**c**) Boxplots showing 5′-3′ delta-Cq (dCq) values calculated from a 5′ and a 3′ RT-qPCR assay of 7 RNA samples isolated from 20,000 FACS sorted *fli1a:EGFP* zebrafish cells with the RNAqueous micro (*left*) and the RNeasy plus micro (*right*) kit. Red dots represent 5′-3′ dCq values of individual samples. * *P* < 0.05 (Wilcoxon sum rank test). (**d** )Average Cq values of a qPCR assay for *elfa* and *loopern4* of 5 RNA samples (2 ng input) purified with the RNAqueous micro or the RNeasy plus micro kit. In total, four different gDNA removal strategies were evaluated: 1) no DNase treatment, 2) gDNA removal strategy of the RNA isolation kit 3) Heat & Run DNase treatment or 4) gDNA removal from the kit + heat & run DNase treatment)

Evaluation of RNA integrity was based on RQN score (ranging from 0 to 10) measurements by means of capillary electrophoresis using the Fragment Analyser (Advanced Analytical). RNA isolation of 20,000 sorted cells resulted in a low RNA yield (see further), making it difficult to measure the obtained RNA yield with frequently used devices such as using the Nanodrop or Qubit. Therefore, the Fragment Analyzer was also used for RNA concentration determination. Based on the RQN values, both kits enable purification of intact RNA with a mean RQN score of 9.45 (range: 8–9.7) for the RNAqueous micro kit and 9.45 (range: 8.5–9.7) for the RNeasy plus micro kit (Fig. 1a). The median yield of the samples isolated with the RNAqueous micro kit (18.5 ng, ranging from 14.5 ng to 41.11 ng) is lower (but not significant) than the RNeasy plus micro kit (28.3 ng, ranging from 20.98 ng to 30.43 ng), while the latter kit also seems more robust, with lower inter-sample variability (Fig. 1b).

Since RQN calculations are mainly based on the integrity of ribosomal RNAs, we additionally performed an RT-qPCR based approach to further assess the mRNA quality of the samples. When using oligo-dT primers to initiate reverse transcription of RNA, the cDNA synthesis reaction starts from the 3′ polyA tail and proceeds to the 5′ end of the mRNA transcript. Therefore, in case of fragmented mRNA, cDNA synthesis will be interrupted, resulting in a lower 5′/3′ relative quantity ratio (equivalent to higher 5′-3′ delta-Cq values). We designed 2 RT-qPCR assays, one targeting the 5′ end and one targeting the 3′ end of the *hprt1* reference gene [20]. We performed RT-qPCR analysis for both the 5′ and the 3′ assay on 7 samples per kit with similar RQN values and calculated the 5′ – 3′ delta-Cq values. The obtained delta-Cq values for both kits were noticeable low (< 1.16), thus indicating a high ‘molecular’ integrity of the isolated RNA. Yet, a significantly lower delta-Cq was observed for the RNAqueous micro kit (median delta-Cq = 0.62, range: 0.37–0.84) compared to the RNeasy plus micro kit (median delta-Cq = 0.89, range: 0.78–1.17) indicating that the highest level of intact RNA is obtained with the RNAqueous micro kit (Mann-Whitney test, *p*-value = 0.0023) (Fig. 1c).

These results indicate that both RNA isolation kits work well for RNA isolation of a low number of sorted cells. RNA with comparable high RQN values and yield is obtained. However, when evaluating RNA quality based on 5′-3′ delta Cq values, the RNAqueous micro kit performs slightly better, indicating less mRNA degradation.

### Additional genomic DNA removal step to further improve RNA quality for RNA-sequencing

Both tested RNA isolation methods incorporate a genomic DNA (gDNA) elimination step during extraction. The RNeasy plus micro kit works through a ‘gDNA eliminator’ spin column, while the RNAqueous micro kit provides reagents for an optional post-elution DNase step. Since gDNA contamination can lead to a bias in gene expression measurements, we tested whether the DNase treatment supplied by the RNA isolation kit was sufficient or whether an additional gDNA elimination step was required. For each kit, 5 RNA samples were isolated following standard procedure with the gDNA elimination step from the kit and for 5 RNA samples no gDNA removal step was carried out. The resulting RNA was split into two samples. Half of the volume was treated with an additional gDNA removal step (Heat & Run) while the other half was used in the next step without additional manipulations. Heat & Run (Articzymes) DNase treatment was chosen as additional gDNA removal step because it does not require an additional purification step in which RNA could be lost.

We used 2 ng of this purified RNA form 1 representative embryo as input for a qPCR reaction with primers for a classic reference gene (*elfa*) and the expressed repeat element (ERE) *loopern4*, the latter recently proposed as a robust reference repeat [22, 23] for gene expression normalization in zebrafish expression studies. Since RNA was used as input (which is not suitable as template for a DNA polymerase), the obtained Cq value is a measurement for the presence of residual gDNA. Using this strategy we could measure the difference in gDNA contamination associated with each of the tested gDNA removal strategies: (1) no DNase treatment, (2) gDNA removal strategy of the RNA isolation kit, (3) Heat & Run DNase treatment or (4) gDNA removal from the kit together with heat & run DNase treatment. We observed that RNA isolated with both kits contained a substantial amount of residual gDNA indicating that a gDNA removal step is required (Fig. 1d). Since EREs are multi-copy repeats in the genome, we expected to observe a lower Cq value compared to a regular reference gene.

For the RNeasy plus micro kit, the gDNA elimination step provided by the kit is very effective with hardly any gDNA being detected. Only when using primers targeting the *loopern4* repeat (ERE), gDNA contamination was noted, indicating that this is only a limited amount and no additional Heat&Run gDNA removal step is required. Yet, for the RNAqueous micro kit, the gDNA elimination step provided by the kit is not sufficient and an additional gDNA removal step is required. When combining the gDNA removal procedure provided by the kit together with Heat&Run DNase treatment, most but not all of the contaminating gDNA could be removed (Fig. 1d, Additional file 2 for statistics).

Just as shown in the manual of the kit, we observed a minimal RNA loss when performing an additional Heat & Run gDNA removal step (data not shown).

Taken together, since gDNA contamination could bias gene expression studies [24, 25], it is a recommended to build in and additional gDNA removal step such as Heat&Run (Articzymes) when using the RNaqueous micro kit.

### Sorting small cell populations directly into the lysis buffer of the RNA isolation kit enhances RNA integrity

FACS sorting is a stressful process that may reduce cell viability and subsequently the quality of the isolated RNA. To overcome this problem, we tested whether sorting directly into the lysis buffer could preserve RNA quality. However, a critical consequence of this approach is dilution of the lysis buffer by the FACS buffer thus possibly influencing its lysis potential as well as the obtained RNA yield and quality. To investigate this, we analysed the maximal diluting factor of each lysis buffer with retention of its lysing capacity. We sorted a range of cells (5000–200,000 cells) in either a collection medium or in the recommended volume of the lysis buffer for both kits and compared both RNA yield and RQN values (quality indication).

**Fig. 2:**
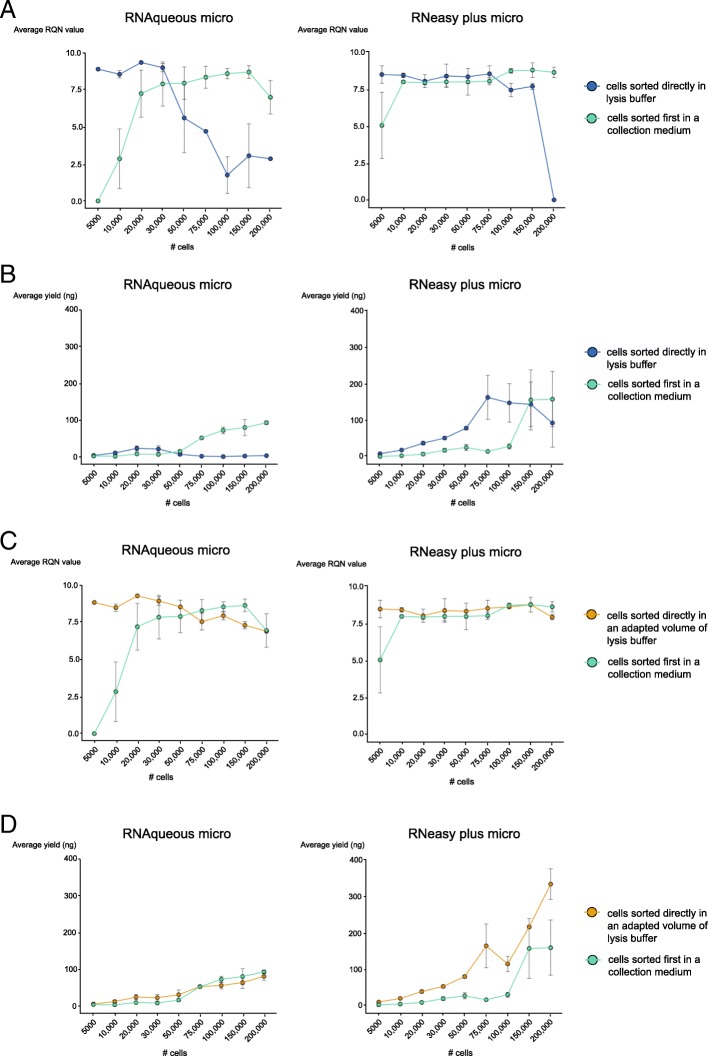
Sorting small cell populations directly into the lysis buffer of the RNA isolation kit improves RNA quality and yield. (**a**) RQN values (**b**) and RNA yield of RNA samples isolated from a range of cell numbers (5000–200,000) when sorting directly into the lysis buffer of the RNA isolation kit or collecting the cells first into a collection medium. RQN values (**c**) and RNA yield (**d**) of RNA samples isolated from a range of cell numbers (5000–200,000) sorted directly into the lysis buffer or sorted into a collection buffer first. But here the volume of the lysis buffer was amended not to exceed its maximum dilution point caused by the sorting procedure. Left panels show samples isolated with the RNeasy plus micro kit, right panels with the RNAqueous micro kit. Average of two biological replicates is shown. Error bars indicate SEM

For the RNAqueous micro kit, based on RQN comparison, sorting into the lysis buffer was beneficial up to a sort volume of 146 μl (=maximum diluting point), which is equal to 30,000 cells in the tested sorting set-up. This volume roughly corresponds to the used volume of the lysis buffer (100 μl), indicating that the lysis buffer could be diluted to roughly 0.5× its volume without loss of its lysis potential. Sorting up to 30,000 cells (146 μl) resulted in a better RNA quality (higher RQN) when sorting directly into the lysis buffer compared to sorting into collection buffer. Of note, with increasing cell numbers, the lysis buffer is further diluted, resulting in lower RQN values (Fig. 2a).

The RNeasy plus micro kit uses a higher recommended lysis buffer volume than the RNAqueous kit and consequently the turning point for switching to sorting into a collection medium is much higher. Here, sorting up to 75,000 cells in a volume of 360 μl resulted in higher RNA quality when sorting into the lysis buffer. The volume of the sorted cells corresponds again roughly to the used volume of the lysis buffer (350 μl), indicating that the lysis buffer keeps its lysing potential up to 0.5× dilution (Fig. 2a).

In addition to the positive effect on RNA quality obtained by sorting directly into the lysis buffer, the yield is considerably enhanced by avoiding a centrifugation step for a low number of cells (Fig. 2b). To keep the yield high, we tested whether increasing the amount of lysis buffer would result in high quality RNA when sorting a larger number of cells. The amount of lysis buffer used was doubled when the maximum tolerated dilution was reached. For example, 100 μl of the RNAqueous kit can cope with 146 μl of cells without loss of its lysis potential. Thus, when sorting more than 146 μl of cells another 100 μl of lysis buffer was foreseen in its collection tube. For the RNeasy plus micro kit, every multiple of 350 μl of sorted cells was collected in a multiple of 350 μl of lysis buffer. The exact volume of lysis buffer used for each cell number can be found in Table 1.

**Table 1:** Volume of lysis buffer used when its volume was adapted to the amount of sorted cells

RNA isolation kit	# sorted cells	Volume of sorted cells (average)	Volume of lysis buffer used to collect sorted cells
RNAqueous micro	5000	24.3 μl	350 μl
	10,000	48 μl	350 μl
	20,000	97 μl	350 μl
	30,000	146 μl	350 μl
	50,000	240 μl	350 μl
	75,000	360 μl	350 μl
	100,000	480 μl	700 μl
	150,000	730 μl	700 μl
	200,000	970 μl	1050 μl
RNeasy plus micro	5000	24.3 μl	100 μl
	10,000	48 μl	100 μl
	20,000	97 μl	100 μl
	30,000	146 μl	200 μl
	50,000	240 μl	300 μl
	75,000	360 μl	400 μl
	100,000	480 μl	500 μl
	150,000	730 μl	700 μl
	200,000	970 μl	900 μl

We could confirm that by keeping the lysis buffer below its maximum diluting point (0.5×), the RNA quality remained high (RQN of > 7.5) and in the case of the RNeasy plus micro kit even equally high as cells sorted into collection buffer (Fig. 2c & d).

When comparing the average yield and RQN, we conclude that sorting up to 75,000 cells into 350 μl of lysis buffer from the RNeasy plus micro kit is beneficial, compared to sorting into 350 μl collection buffer. In addition, when correcting the volume of lysis buffer to avoid dilution and reduction of RNA quality, the RNA yield when sorting into lysis buffer stays higher than sorting into collection buffer (Fig. 2c). For the RNAqueous micro kit, sorting into the lysis buffer was only beneficial for yields up to 30,000 cells. Beyond this cell count, sorting into collection buffer is advised. Correction of the used amount of lysis buffer did not further improve yield and quality. Here both yield and RQN values are equal or higher in the samples sorted into collection buffer when sorting more than 30,000 cells (Fig. 2c & d).

It is important to note that the volume of the sorted cells is defined by the type of cell sorter that was used, for example, the FACS that was used here (Bio-Rad S3e) sorts on average 1,000,000 cells in a volume of 4,8 ml FACS buffer. Both lysis buffers could endure roughly a 0.5× dilution, the number of cells correspond to this sorted volume is defined by the chosen type of cell sorter and should therefore be determined in advance.

In view of these data, we can conclude that sorting a small cell number directly into the lysis buffer of the RNA isolation kit is beneficial for RNA quality and yield up to 0.5× dilution of the lysis buffer. When sorting a higher volume (and thus a higher cell number) it is advised to either adapt the lysis buffer volume or sort into a collection medium first and then pelleting the cells before adding lysis buffer.

### Sorting cells into the lysis buffer preserves and protects the RNA from degradation

Given that sorting based isolation of fluorescent cells induces a stress response and that RNA isolation directly after sorting might not always be feasible, we analysed the effect of the time interval between cell sorting and RNA isolation on the quality of the obtained RNA.

**Fig. 3:**
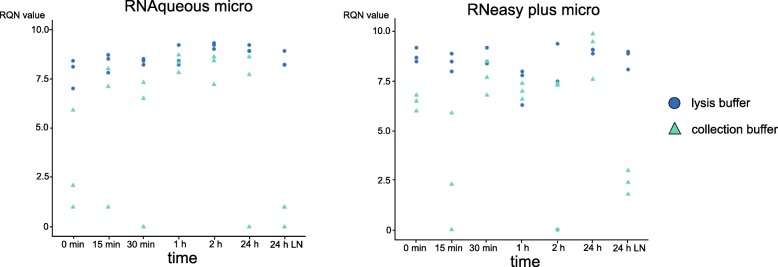
Sorting cells directly into the lysis buffer preserves and protects RNA from degradation. RQN values of individual RNA samples isolated at specific time points after FACS sorting of 20,000 *fli1a:EGFP* cells. Cells were sorted directly in the lysis buffer of the kit or first captured into a collection medium. Left panel shows samples isolated with the RNAqueous micro kit, right panel with the RNeasy plus micro kit

We isolated RNA at different time points (0 min, 0.15 min, 30 min, 1 h, 2 h, 24 h, and frozen in liquid nitrogen for 1 week) post FACS sorting of 20,000 cells with both RNA isolation methods and assessed RNA integrity with the Fragment Analyser. Since sorting into the lysis buffer of the kit will directly lyse the cells and protect the released RNA, we investigated whether the RNA integrity could be preserved for a longer period of time compared to sorting into a collection buffer. RNA was isolated directly after sorting or after the cells were kept for 15 min, 30 min, 1 h, 2 h or 24 h on ice. In addition, samples were frozen into liquid nitrogen and kept at − 80 °C for 1 week to evaluate if longer storage was possible. The time between cell sorting and RNA isolation did not influence RNA integrity for both kits. However, sorting directly into the lysing buffer of the RNA isolation kit consistently resulted into high(er) quality RNA. In contrast, when sorting into a collection buffer, the RNA quality was highly variable and the RQN ranged from 0 and 10. Obtaining high-quality RNA after long-term storage or a longer period of time between sorting and subsequent RNA isolation was only possible when the RNA was protected by the lysis buffer from the kit (Fig. 3).

This shows that sorting directly into the lysis buffer preserves and protects the RNA from degradation up until RNA isolation, thus allowing to retrieve high quality RNA.

### PolyA+ RNA sequencing

After successful isolation of high quality RNA, we tested whether the SMART seq V4 cDNA synthesis followed by Nextera XT library preparation could be used for successful sequencing of our samples. In a first step, we used RT-qPCR to determine the expression of certain key genes in GFP positive versus GFP negative sorted *fli1a:EGFP* cells. For each RNA isolation kit, 3 × 20,000 GFP positive and 3 × 20,000 GFP negative cells were sorted directly into the lysis buffer of the RNA isolation kit. Heat & Run was performed to completely clear the RNA from contaminating gDNA and cDNA was synthesized with the SMART seq V4 kit. RT-qPCR analysis clearly shows *fli* and *EGFP* expression in the EGFP positive cell population, whereas no (or negligible) expression in the EGFP negative population (Additional file 3: Figure S2A).

**Fig. 4:**
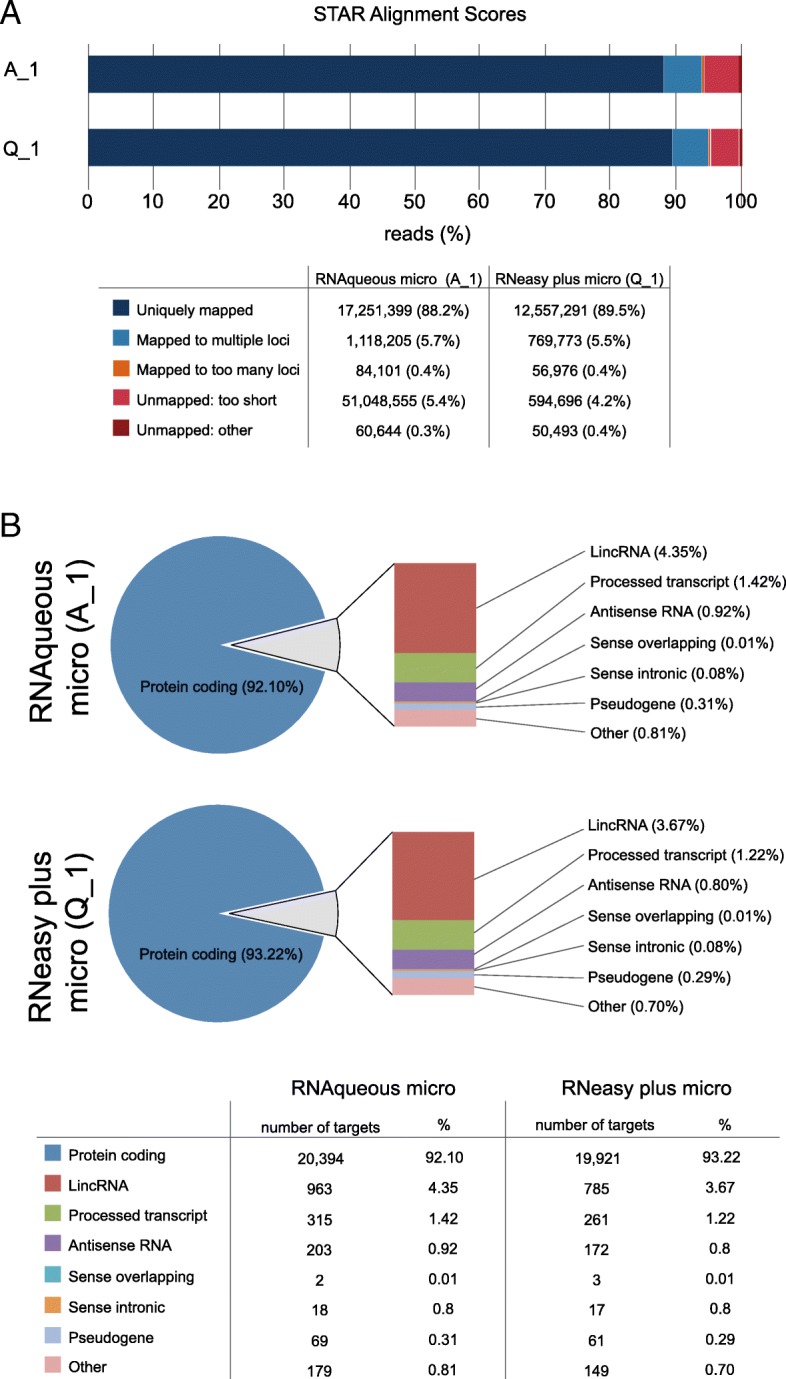
PolyA RNA sequencing on sorted zebrafish cells. (**a**) Star alignment scores indicate percentage of (uniquely) mapped reads. (**b**) Distribution of the different biotypes that were detected with our polyA RNA sequencing protocol. The biotype indicated as ‘Other’ includes: misc_RNA, Mt_rRNA, TR_V_gene, scaRNA, sRNA, snoRNA, rRNA, miRNA, mt-tRNA, TEC and snRNA

Subsequently, to test whether SMART seq V4 cDNA synthesis was suitable for RNA sequencing, one initial sample for each RNA isolation kit, sample A_1 (RNAqueous micro) and sample Q_1 (RNeasy plus micro), was processed using the same work flow but now Nextera XT library prep was performed on the SMART seq V4 generated cDNA. These two initial samples were then sequenced using the Illumina Nextseq500 to get a first idea whether this workflow fits our purposes (GSE121917). Both RNA isolation kits resulted in a high percentage of uniquely mapped reads (89.5% for the RNAqueous micro kit and 88.2% RNeasy plus micro) and FastQC revealed good sequencing quality (Fig. 4a). In both samples, roughly 90% of the reads mapped to known protein coding genes. A detailed overview RNA biotype distribution is shown in Fig. 4b.

To test reproducibility of our applied method, additional samples were processed (GSE121917). For each RNA isolation kit, 1 RNA sample was split to generate 2 technical replicates (samples A_2a, A_2b, Q_2a and Q_2b) and they were treated as individual samples for cDNA synthesis, library prep and sequencing. For sample details (Table 2). All four samples had roughly 85% uniquely mapped reads with less than 0.5% difference between the two technical replicates. Mapping percentages and biotype distribution of all samples can be found in Table 3 and Table 4.

**Table 2:** Sample specifications and input details for SMART-seq V4 cDNA synthesis and amplification and for Nextera XT library prep

sample	RNA isolation kit	# cells	RNA Concentration	RQN	input SMART-seq v4	# amplification cycles	cDNA concentration	input cDNA Nextera XT
A_1	RNAqueous	20,000	0.75 ng/μl	9.6	9.5 μl	11	0.476 ng/μl	1 ng
Q_1	RNeasy	20,000	1.20 ng/μl	9.6	9.5 μl	11	1.24 ng/μl	1 ng
A_2a	RNAqueous	20,000	0.79 ng/μl	9.2	6 μl	13	13.6 ng/μl	1 ng
A_2b	RNAqueous	20,000	0.79 ng/μl	9.2	6 μl	13	15.6 ng/μl	1 ng
Q_2a	RNeasy	20,000	1.02 ng/μl	9.6	6 μl	13	16.1 ng/μl	1 ng
Q_2b	RNeasy	20,000	1.02 ng/μl	9.6	6 μl	13	16.2 ng/μl	1 ng
A_unsorted	RNAqueous	1 embryo	1.5 ng/μl	10	9.5 μl	11	3.06 ng/μl	1 ng
Q_unsorted	RNeasy	1 embryo	1.5 ng/μl	10	9.5 μl	11	5.95 ng/μl	1 ng

All samples, except A_unsorted and Q_unsorted, are 20,000 EGFP positive sorted cells from the same pool of Tg*(fli1a:EGFP)* zebrafish and RNA concentration was measured with the Fragment Analyzer. RNAqueous 2a and 2b and RNeasy 2a and 2b are technical replicates, starting from the same RNA. A_unsorted and Q_unsorted samples were obtained by isolating RNA from 1 embryo, RNA was measured with the Nanodrop (ThermoScientific) and RNA was diluted to 1.5 ng/μl to start with roughly the same input. cDNA concentration of all samples was measured with the Qubit dsDNA HS Assay kit (Invitrogen)

**Table 3:** Star alignment mapping of the sequenced samples

	A_1	A_2a	A_2b	A_unsorted	Q1	Q_2a	Q_2b	Q_unsorted
uniquely mapped	88.18%	85.99%	84.29%	83.94%	89.51%	85.49%	85.84%	79.62%
mapped to multiple loci	5.72%	6.70%	6.60%	6.32%	5.49%	6.93%	6.72%	11.12%
mapped to too many loci	0.43%	0.19%	0.22%	0.24%	0.41%	0.19%	0.19%	0.23%
unmapped: too short	5.36%	6.83%	8.57%	9.21%	4.24%	7.14%	7.01%	8.69%
unmapped: other	0.31	0.28	0.31	0.30	0.36	0.24	0.24	0.34

Summary table of the star alignment mapping scores showing percentages of mapped reads per sample. High percentage of uniquely mapped reads is obtained in all samples. Samples starting with an “A” are samples where RNA was isolated with the RNAqueous micro kit. Samples starting with a “Q” are samples where RNA was isolated with the RNeasy plus micro kit. A_1 and Q_1 were sequenced on a different time point then the other samples. No differences were seen between the two RNA isolation kits

**Table 4:** Biotype distribution of sequenced samples

	A1	A_2a	A_2b	A_unsorted	Q1	Q_2a	Q_2b	Q_unsorted
protein coding	92.101%	96.242%	96.616%	95.606%	93.224%	96.067%	95.711%	98.159%
linRNA	4.349%	2.113%	2.154%	1.945%	3.674%	1.918%	2.014%	0.579%
processed transcript	1.423%	0.255%	0.215%	0.287%	1.221%	0.280%	0.297%	0.304%
antisense RNA	0.917%	0.027%	0.025%	0.035%	0.805%	0.026%	0.027%	0.019%
sense_overlapping	0.009%	0.000%	0.000%	0.000%	0.014%	0.000%	0.000%	0.000%
sense_intronic	0.081%	0.002%	0.002%	0.003%	0.080%	0.002%	0.002%	0.002%
pseudogene	0.312%	0.024%	0.023%	0.027%	0.285%	0.024%	0.022%	0.026%
other	0.808%	1.315%	0.945%	2.053%	0.697%	1.665%	1.906%	0.859%

Summary table showing the biotype distribution (in percentage) of the sequenced samples. More than 90% of the processed reads are protein

**Fig. 5:**
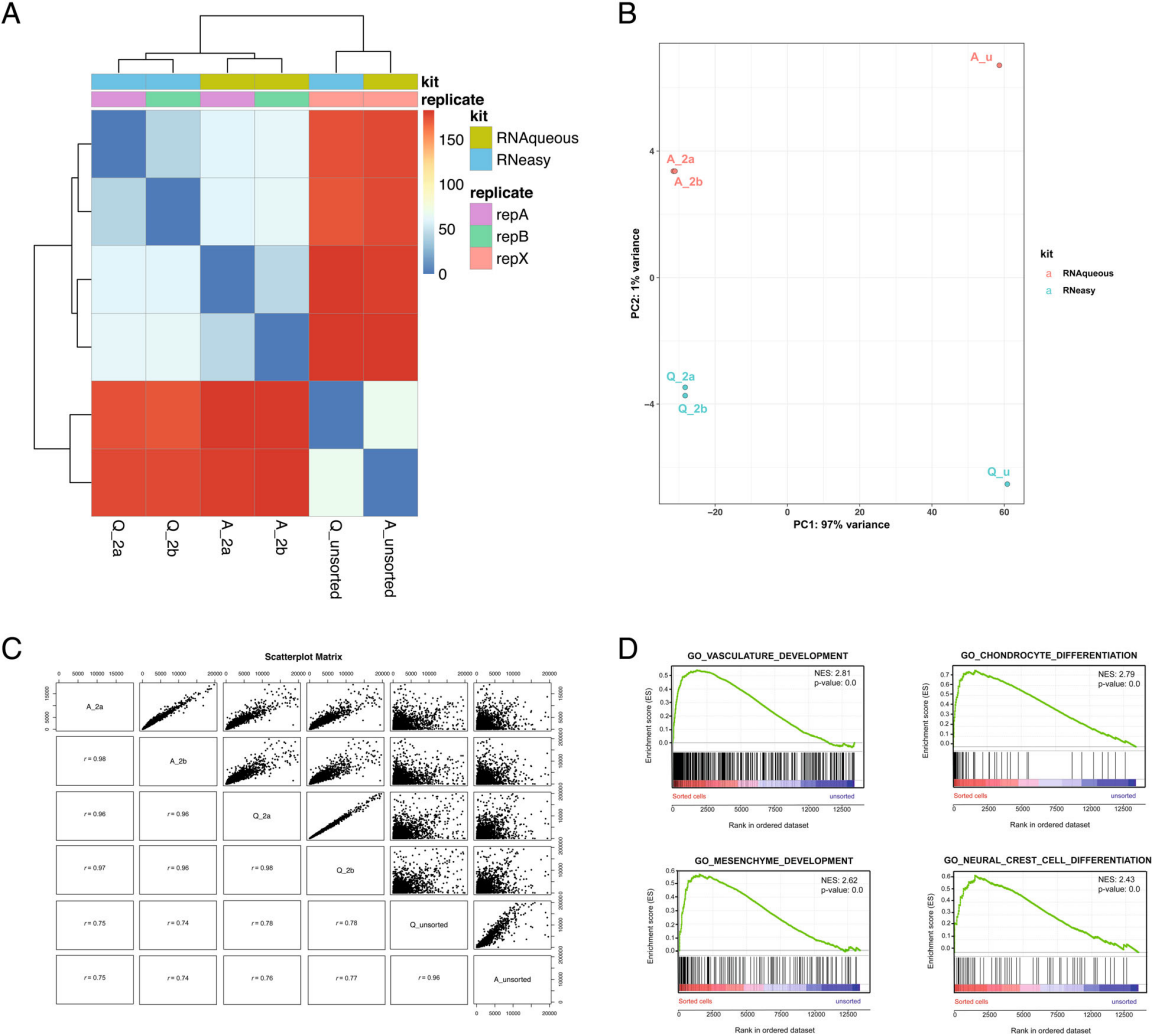
Reproducibility analysis of the presented polyA RNA sequencing workflow. (**a**) Heatmap and (**b**) PCA analysis representing the variation between sequenced samples. Hierarchical clustering and PCA clustering demonstrate that the technical replicates (Q_2a & Q_2b and A_2a & Q_2b) are the most similar and cluster together. More variance is seen between the sorted samples isolated with different kits (A_2 vs Q_2). All sorted samples cluster away from the unsorted samples. (**c**) Correlation of all the different samples. Pearson’s correlation coefficient *r* is calculated on normalized (Deseq2 normalization) log transformed read counts. (**d**) GO annotated gene sets GO_vasculature_development, GO_chondrocyte_differentiation, GO_mesenchyme_developemnt and GO_neural_crest_cell_differentation are enriched in sorted cells versus unsorted cells

In addition to the technical replicates for each RNA isolation kit, we sequenced RNA isolated from whole embryo samples (unsorted) with both RNA isolation kits to compare with sorted samples. To look at reproducibility of the kit, we performed hierarchical clustering and PCA analysis. Both analyses clearly show that both technical replicates cluster closely together and that all the sorted samples cluster closer to each other than the unsorted whole embryo samples (Fig. 5a & b). When introducing the samples from the first sequencing run (A_1 and Q_1), all the sorted samples from run 2 cluster together, but away from samples from run 1 regardless their RNA isolation kit. This shows that sequencing libraries in different sequencing runs introduce substantial inter-run variation, larger than the effect of the RNA isolation kit. The unsorted samples cluster together and away from the sorted samples (Additional file 4: Figure S3).

A very high correlation, as quantified by the Pearson’s correlation coefficient, was noted for the technical replicates (*r* = 0.98 both kits) (Fig. 5c). The correlation between sorted samples isolated with different RNA isolation kits is also high (*r* > 0.96) and the 2 two unsorted samples (*r* = 0.96). The introduced variation could be partly due to the RNA isolation kit or due to the difference in the sorted cell population since *fli1a* is expressed in different cell types. As expected, sorted cells did not correlate well with unsorted whole embryos (*r* < 0.74). All the above-presented data shows that our applied method generates good quality data and is highly reproducible.

The *fli1a* gene is a DNA binding transcription factor that is involved in vascular development. Expression is observed in neural crest derived cells such as developing cartilage in the jaw, vasculature, and developing mesenchyme. Gene Ontology (GO) annotations linked to the *fli1a* zebrafish gene include DNA binding transcription factor activity (RNA polymerase II specific), angiogenesis, blood vessel morphogenesis, regulation of transcription by RNA polymerase II, and cell differentiation. Gene set enrichment analysis (GSEA) was performed on unsorted (whole embryo) versus *fli1a* + sorted cells to see if the same GO annotated gene sets were enriched in the sorted *fli1:GFP* sampes compared to the unsorted samples. This was indeed the case, indicating that sorting enriched for *fli1a* expressing cells. Gene sets like GO_vasculature, GO_cartilage_development and GO_mesenchyme_development were enriched in the EGFP sorted cells (Fig. 5d).

To further validate our method, we tested the workflow on different transgenic lines and confirmed with RT-qPCR the expression of key genes for the specified cell population. We sorted thymocytes from Tg(*rag2:GFP*) embryos and were able to show expression of tissue specific genes through RT-qPCR analysis. Expression of *lck* (thymocyte specific gene) and *GFP* is detected in the GFP positive samples but not in the GFP negative samples (Additional file 3: Figure S2B). In addition, we successfully used our workflow on sorted tumor cells from Tg(*rag2:mMyc;rag2:GFP)* or Tg(*dβh:MYCN, dβh:GFP)* adult zebrafish (data not shown).

These results demonstrate that SMART-seq V4 followed by Nextera XT is a suitable workflow for polyA+ sequencing of RNA isolated with the RNeasy plus micro kit or the RNAqueous micro kit from a small number of sorted zebrafish cells. A detailed graphical overview and protocol can be found in supplementary files (Additional file 5) to guide your own experiments.

## Discussion

Zebrafish is becoming increasingly popular as an in vivo model for the study of human diseases [26, 27]. Main advantages include rapid development and short generation time, large offspring, transparent embryos and adults, and a rapidly growing toolbox for gene modulation (knockout, gene editing or gene overexpression). The zebrafish genome has been investigated thoroughly and a high quality and precisely annotated reference genome is now available with about 70% of human genes showing at least 1 zebrafish orthologue thus offering interesting prospects for functional genomic analyses [28].

Various analytical methods including RT-qPCR and RNA sequencing can be applied for the evaluation of gene expression in specific cell types or to analyse expression changes upon a specific genetic or chemical perturbation. RNA-sequencing has become the preferred method for transcriptome-wide analysis offering multiple advantages to hybridization-based approaches, such as higher sensitivity, dynamic range and insights into novel transcribed regions, alternative splicing and allele-specific expression [29–32].

One of the powerful features of the zebrafish model is the possibility to use transgenic reporter lines for the isolation of specific cell types through fluorescence-based cell sorting and subsequent tissue and cell specific transcriptome analysis on in vivo samples [13–15, 33, 34]. Of note, tissue dissociation and cell sorting may induce cellular stress responses that influence cell viability and quality of the isolated RNA [17]. As RNA degradation does not occur at the same rate or to the same extent for every transcript, RNA integrity impacts expression studies highlighting the importance of RNA quality assessment. In addition, when sorting a specific cell type from zebrafish embryos, sample size might be limited to only a few thousand cells. RNA isolation and sequencing of a low number of sorted cells remains challenging. Since most of the available protocols are focused on either a high number of cells or specifically adapted for single cells [32, 35, 36], we here optimized a workflow for RNA isolation and sequencing of a low number (5000–100,000) of sorted zebrafish cells. This protocol could be especially useful when the cells of interest are only available in low numbers. In addition, we incorporated an in depth quality assessment that could serve as an example for other sequencing experiments and experimental set ups.

We compared the performance of two RNA isolation kits for obtaining high-quality RNA from FACS sorted cells, i.e. the RNAqueous Micro Total RNA Isolation Kit from Ambion and the RNeasy Plus Micro Kit from Qiagen. Based on different quality parameters and comparison of various experimental set-ups, we recommend using the RNeasy Plus Micro Kit for RNA isolation of FACS sorted zebrafish cells. The RNA purification protocol is fast (only 20–30 min), easy and consistently delivers high-quality RNA samples. In addition, sorting into the lysis buffer will protect RNA up until the moment of purification, making it possible to postpone RNA isolation after sorting.

To maximize the RNA quality and yield, direct sorting into the lysis buffer from the RNA isolation kit is advised. In this way, the sorted cells will be lysed immediately and the released RNA will be protected from degradation. However, as the sorted cells reside in sorting buffer, dilution of the lysis buffer reduces the lysis potential. Therefore, sorting directly into the lysis buffer is only beneficial up to a certain number of sorted cells. This maximum cell number is different for each RNA isolation kit used. Both RNA isolation kits yield good quality RNA up to a 0.5 times dilution of their respective lysis buffers. For the sorter (Bio-Rad S3e) used in our experiments, this corresponds with 30,000 cells for the RNAqueous micro kit and 75,000 cells for the RNeasy plus micro kit.

Sorting into a RNA protecting agent, like RNA*later* (Ambion), could also help stabilizing the RNA after sorting and thus prevent RNA degradation [17]. As RNA*later* is more viscous, pelleting by spin down of at least 30 min is required to recovering the cells for RNA isolation. When working with a small number of cells, this pelleting step may lead to cell loss during this procedure. In addition, when sorting into RNA*later*, you will dilute this protecting agent. Similar as to sorting into lysis buffer, this requires analysis of how much dilution can be endured without the risk of losing its protecting capacity.

After successful isolation of high quality RNA, an additional gDNA elimination step can be incorporated into the workflow to completely eliminate residual gDNA in the RNA sample. We could show that the gDNA elimination step from the RNaqueous micro kit does not sufficiently remove contaminating gDNA. To fully purify the RNA from residual gDNA, an additional DNA removal step is required. For the RNeasy plus micro kit, the gDNA elimination step provided by the kit is most efficient to remove contaminating gDNA. Yet, this was tested on low input material (two embyros) so when more input is provided, the gDNA removal column might be saturated and gDNA removal will be insufficient. Thus, it is advised to test gDNA contamination for your chosen experimental set up. The presence of gDNA in a RNA sample may lead to overestimation of the abundance of certain transcripts and thus causes bias in expression studies [24, 25]. The Heat&Run kit (ArticZymes) is especially useful for gDNA removal because it does not involve an additional purification step where the already sparse RNA could be lost. The obtained RNA, free of gDNA, could subsequently be used for RNA sequencing by using SMART-seq v4 for cDNA synthesis and amplification and Nextera XT for library prep. This experimental workflow successfully generated highly reproducible RNA sequencing data.

The SMART seq v4 kit is suitable for RNA sequencing up to single cell level. When sorting a single cell into the SMART seq lysis buffer, the cell will be lysed immediately and cDNA synthesis and amplification can be performed directly on the cell lysate [31]. The resulting cDNA can then be used as input for Nextera XT library preparation. When working with higher number of cells (in this study 5000–100,000), sorting into the lysis buffer of the SMART seq v4 kit will dilute the buffer to the extent that it loses its lysis potential. Sorting into a collection medium, pelleting the cells and adding the lysis buffer is unfavourable for both quality and yield and did not work well in our hands. To overcome this problem, we incorporated an RNA isolation step in our workflow with SMART seq v4 performed on the purified RNA to generate and amplify cDNA.

The proposed workflow is currently applicable for polyA RNA-sequencing of small (5000–50,000) numbers of sorted zebrafish cells. In addition to polyA tailed transcripts, representing roughly one third of the entire transcriptome, many other non-polyadenylated RNAs are expressed including a wide variety of long non-coding RNAs. In principle, this can be achieved using a total RNA-sequencing workflow, but this requires a ribosomal RNA depletion step which is not yet available for zebrafish [29, 37].

## Conclusions

In conclusion, we optimized a workflow for polyA+ RNA sequencing of a low number (5000–100,000) of sorted zebrafish cells. We identified that sorting the cells directly into the lysis buffer of the kit maximizes the quality and the yield of the RNA. Yet, this is only beneficial up to a certain volume of sorted cells due to dilution of lysis buffer. For both RNA isolation kits, we could show that the lysis buffer could endure 0.5× dilution without losing its lysis potential and lowering the RNA quality and yield. When sorting a higher number of cells, we advise to increase the lysis buffer volume or to sort into a collection medium first. After isolation, the quality of the isolated RNA should always be assessed before proceeding to the next step. Incorporation of an additional gDNA removal step (for example with the Heat&Run kit, ArticZymes) is advised to completely clear the RNA from contaminating gDNA. Full length cDNA synthesis and amplification can be done by Smart seq v4 followed by tagmentation and sample barcoding by Nextera XT. This workflow enables to retrieve high quality RNA from FACS sorted zebrafish cells and yields highly reproducible RNA sequencing data. Since RNA quality is critical when performing transcriptome studies, we strongly recommend to do an in depth quality assessment of the isolated RNA as presented in this study, when implementing a new RNA sequencing workflow.

## Material & Methods

### Zebrafish maintenance

Zebrafish were maintained in a zebtec semi-closed recirculation housing system (Techniplast, Italy) with a constant water temperature of 28 °C and a conductivity of 500 μS. The fish are exposed to a daily 14 h light and 10 h darkness cycle and fed 2 times/day with dry food (SDS, UK) and once with Artemia (Ocean Nutrition). The Tg(*fli1a:EGFP*) zebrafish were obtained from the zebrafish international resource center (ZIRC). The Tg(*fli1a:EGFP*) were crossed and embryos were collected. The clutch of embryos (50–150) were transferred to a petri dish with E3 media and kept in an incubator at a temperature of 28 °C and a daily light and darkness cycle (see higher) until the moment of further processing. For all our experiments, roughly 10 times 100 embryos were used for FACS sorting. All embryos used were younger than 120 h post fertilization (hpf), and thus no ethical approval was needed.

### Embryo dissociation for FACS sorting

A clutch of 50–150 embryos of age 3–5 days post fertilization (dpf) was euthanized using an overdose of tricaine and transferred to a 35 mm culture dish. The tricaine was removed as much as possible and 3 ml of preheated (28 °C) dissociation solution (1× PBS, trypsin 0.25%, 1 mM EDTA) was added. During an incubation period of 90 min at 28 °C, the larvae dissociation was advanced by pipetting up and down with a 1 ml pipette every 15 min. When the larvae were completely dissociated, the reaction was stopped by adding 3 μl 1 M CaCl_2_ (final concentration 1 mM) and 300 μl FBS (10% final concentration). The cells were transferred to a 15 ml tube, pelleted (5 min 800 g), washed with PBS and resuspended in 2 ml resuspension solution (Leibovitz’s L-15 medium + L-Glutamine without Phenol Red, FCS 10%, 0.8 mM CaCl_2_). The cell solution was passed several times through a 40 μM mesh filter prior to cell sorting. This cell suspension was analysed by flow cytometry on a Bio-Rad S3e cell sorter. Forward and side scatter was used to gate for live, single cells. GFP positive cells, which ranged between 5 and 15% of the parental population, were sorted and collected. No other markers were used for flow cytometry. Representative samples (a negative control vs. GFP positive cells) with the gating strategy are shown in the supplemental data (Additional file 6: Figure S4).

The sorted cells were collected in either the lysis buffer of the RNA isolation kit or into a collection medium (the used resuspension buffer). The cells were kept on ice during the whole procedure.

### RNA isolation and quality control

RNA from sorted cells was isolated with the use of the RNeasy plus micro kit (Qiagen, 74,034) or the RNAqueous-Micro Total RNA Isolation Kit (Ambion, AM1931). For the RNeasy plus micro kit, the RLT buffer was supplemented with 2-mercaptoethanol (10 μl per 1 ml RLT) as suggested. The integrity and the concentration of the RNA was analyzed with the Fragment Analyzer (Advanced Analytical Technologies) High Sensitivity RNA Analysis Kit (DNF-472-0500). The PROSize software version 3.0.1.5 determined a RQN RNA integrity score considering the entire electropherogram.

### 5′ – 3′ delta-Cq RNA integrity assay

This RT-qPCR assay aims to assess the integrity of a low abundant reference gene mRNA, *hprt1*, as a representative of all mRNAs in the sample. By using the oligo dT primed reverse transcriptase iScript Select kit (Bio-Rad 1,708,896), the cDNA synthesis starts from the 3′ polyA tail, proceeding to the 5′ end of the mRNA transcript. Upon mRNA degradation, the cDNA synthesis is interrupted and not completed to the 5′ end of the transcript. We designed two RT-qPCR assays, 1 targeting the 5′ end (*hprt1 5′-fw: CGTTTTGCAGTAGCTTGTCAGA; hprt1 5′-rev: ACACCCGCTCTAAGTCAGC)*, and 1 targeting the 3′ end of the *hprt1* cDNA (*hprt1 3′-fw: GAGGAGCGTTGGATACAGA; hprt1 3′-rev: CTCGTTGTAGTCAAGTGCAT)*. The assays were developed using the NCBI ‘pick primer’ tool and efficiencies were determined based on a relative 6-point 4-fold dilution series (32, 8, 2, 0.5, 0.125, 0.032 ng cDNA of a pool of embryos of various ages). The higher the 5′-3′ dCq value, the more mRNA degradation. RNA used for this assay was isolated from 1 embryo using the RNAqueous micro or the RNeasy plus micro kit as described in the manual. Starting from 250 ng RNA, cDNA was synthesized using the oligodT primers of the iScript select cDNA synthesis kit.

For RT-qPCR, 2.5 μl 2× SsoAdvanced SYBR Green supermix (Bio-Rad) was mixed with 5 ng input cDNA and 250 nM (finale concentration) of each forward and reverse primer in a 384 well plate (Bio-Rad) and run on a LightCycler 480 (Roche). Thermocycler conditions were set as follows: 95 °C for 2 min, followed by 44 cycles of 95 °C for 5 s, 60 °C for 30 s, 72 °C for 1 s and finally a melting curve analysis was performed at 95 °C for 5 s followed by 60 °C for 1 min, gradual heating to 95 °C at a ramp-rate of 0.11 °C/s followed by cooling to 37 °C for 3 min. Cq values were exported and RT-qPCR data were analysed with qbase+ version 2.6.1 (Biogazelle).

### Genomic DNA contamination assessment

For the gDNA contamination assay, RNA was isolated from 2 embryos and diluted to 1 ng/μl to match input amount of sorted cells. Five samples for each kit were isolated without including the gDNA elimination step of the procedure, another five samples for each kit were isolated with the standard RNA isolation method including its gDNA elimination step. Each RNA sample was split into two: half of the RNA was subjected to a Heat & Run (ArticZymes 80,200–50, following the guidelines of the kit) gDNA removal step to remove possible contaminating gDNA. The other halve of the sample was used in further steps without extra gDNA removal. The Heat & Run kit enables gDNA removal without the need of an extra purification step, therefore preventing the loss of sparse RNA. qPCR for the reference gene *elfa (elfa-fw: GGAGACTGGTGTCCTCAA; elfa-rev: GGTGCATCTCAACAGACTT*) and the ERE reference gene [22] *loopern4 (loopern4-fw: TGAGCTGAAACTTTACAGACACAT; loopern4-rev: AGACTTTGGTGTCTCCAGAATG)* was performed on 2 ng RNA from each sample to test for contaminating gDNA. As qPCR is not possible using RNA as template, amplification signals the presence of gDNA in the RNA sample. The RNA was mixed with 2.5 μl 2× SsoAdvanced SYBR Green supermix (Bio-Rad) and 250 nM (finale concentration) of each forward and reverse primer in a 384 well plate (Bio-Rad) and run on a LightCycler 480 (Roche). Thermocycler conditions were as follows: 95 °C for 2 min, followed by 44 cycles of 95 °C for 5 s, 60 °C for 30 s, 72 °C for 1 s and finally a melting curve analysis was performed at 95 °C for 5 s followed by 60 °C for 1 min, gradual heating to 95 °C at a ramp-rate of 0.11 °C/s followed by cooling to 37 °C for 3 min. Cq values were exported and RT-qPCR data were analysed with qbase+ version 2.6.1 (Biogazelle).

### Determination of the maximum dilution point of the lysis buffer of the RNeasy plus micro and the RNaqueous micro kit

A clutch of Tg(*fli1a:EGFP*) embryos was dissociated and processed for FACS sorting as described above. A range of cells (5000; 10,000; 20,000; 30,000; 50,000; 75,000; 100,000; 150,000 and 200,000) were sorted either directly into the lysis buffer of the RNA isolation kit or in a collection medium (= the used resuspension buffer: Leibovitz’s L-15 medium + L-Glutamine without Phenol Red, FCS 10%, 0.8 mM CaCl_2_). When cells were sorted in a collection medium, they were centrifuged (5 min at 1200 rmp), supernatant was removed and the pellet was dissolved in the lysis buffer of the RNA isolation kit. RNA was isolated following the manual of the RNA isolation kit. The integrity and RNA concentration was measured with the Fragment Analyzer as described earlier.

### Time series to test RNA preservation capacity of the lysis buffer after FACS sorting

20,000 GFP+ cells from Tg(*fli1a:EGFP*) embryos were FACS sorted following the procedure described above. The cells were either sorted in lysis buffer of the RNA isolation kit or into a collection medium (Leibovitz’s L-15 medium + L-Glutamine without Phenol Red, FCS 10%, 0.8 mM CaCl_2_). The sorted cells were kept on ice for a specific amount of time (0 min, 15 min, 30 min, 1 h, 2 h or 24 h) before RNA isolation was carried out. The cells that were sorted into a collection medium were pelleted (5 min, 1200 rpm) and dissolved in lysis buffer right at the start of the RNA isolation process. In addition, to test if longer storage was possible, samples were frozen in liquid nitrogen and stored at − 80 °C for a week before proceeding to RNA isolation. RNA quality was determined with the Fragment Analyzer as described above.

### cDNA synthesis and RT-qPCR

Following the Heat & Run guidelines, 1 μl HL-dsDNase and 2 μl 10× Rxn buffer (Heat & Run, Articzymes) were added to 20 μl of each RNA sample. Samples were incubated for 10 min at 37 °C and 5 min at 58 °C for gDNA removal. cDNA synthesis and amplification was prepared using the SMART-Seq v4 Ultra Low Input RNA Kit (Takara Biosystems 634,890). cDNA concentrations were measured using the Qubit dsDNA high sensitivity assay kit (Invitrogen, Q32851). Each sample was diluted to 0.5 ng/μl and 2 μl was mixed with 2.5 μl 2× SsoAdvanced SYBR Green supermix (Bio-Rad) and 250 nM (finale concentration) of each forward and reverse primer in a 384 well plate (Bio-Rad) and run on a LightCycler 480 (Roche). Thermocycler conditions were as follows: 95 °C for 2 min, followed by 44 cycles of 95 °C for 5 s, 60 °C for 30 s, 72 °C for 1 s and finally a melting curve analysis was performed at 95 °C for 5 s followed by 60 °C for 1 min, gradual heating to 95 °C at a ramp-rate of 0.11 °C/s followed by cooling to 37 °C for 3 min. Cq values were exported and RT-qPCR data were analysed with qbase+ version 2.6.1 (Biogazelle). The ERE reference genes *trd7*, *loopern4*, *hatn10* were used for normalization. For primer sequences, see Table 5.

**Table 5:** RT-qPCR primer sequences

gene	forward primer	reverse primer
*tdr7*	GCAGCATAATTGAGTACACCC	TTGCCTATATTCACTGAGAAATGGA
*hatn10*	ACTAATGAAGACAGCAGAAGTCA	CAGTAAACATGTCAGGCTAAATAAT
*loopern4*	TGAGCTGAAACTTTACAGACACAT	AGACTTTGGTGTCTCCAGAATG
*flia*	GGGCTCCACTGAAAATTGCG	CTGGCCGTAATCCTGAGTCC
*lck*	ACATGTCTTTGAAACACGCCG	GCAGTTCCCCATGTTTACGTATTT
*GFP*	ACACTGACCAAGAGCTACCTTC	GTTTTGGCCAGCCCTTTTGT
*EGFP*	GACCACATGAAGCAGCAC	TTGTCGGCCATGATATAGAC

### Library prep, sequencing and data analysis

1 ng of SMART-Seq v4 amplified cDNA (Takara Biosystems 634,890) was used for Nextera XT DNA library prep (Illumina, FC-131-1024). For sample and input specifications see Table 2.

Libraries were sequenced using a NextSeq 500 (Illumina). Quality control on fastq files was performed with FastQC (https://www.bioinformatics.babraham.ac.uk/projects/fastqc/).

Reads were aligned to *Danio rerio* reference genome GRCz10 with STAR v2.4.2a using a two-pass strategy. Genes were quantified using the Danio_rerio.GRCz10.91.gtf transcriptome.

The DESeq2 R-package (version 1.20.0) was used for count normalization and differential gene expression analysis. The normalized read counts were used to generate PCA plots, heatmaps and the correlation matrix. Pearson’s correlation coefficient was calculated on log transformed normalized read counts. Pre-ranked Gene Set Enrichment Analysis (GSEA) was preformed using GenePattern 2.0 (https://cloud.genepattern.org/gp/pages/login.jsf).

## Additional files

Additional file 1: **Figure S1**. Adaptation of the RNA isolation protocol allows purification of small RNA’s (< 200 nt). Fragment analyser electropherogram shown for the RNAqueous micro kit (*top*) and the RNeasy plus micro kit (*bottom*) for normal isolation procedure (*left*) and for RNA isolation with the adapted protocol for small RNA’s as provided in the manual (*right*). The red arrow indicates presence of small RNA’s in the isolated RNA sample (PDF 1667 kb)

Additional file 2: Supplementary note 1: Statistical analysis (DOCX 147 kb)

Additional file 3: **Figure S2**. RT-qPCR confirms tissue specific gene expression in tissue specific sorted cells. (A) From a clutch of Tg(*fli1a:EGFP)* embryos, EGFP positive and EGFP negative cells were sorted. RT-qPCR analysis shows expression of *EGFP* and *fli1*a is in the EGFP positive samples but not or negligible in the EGFP negative samples. (B) RT-qPCR analysis confirms GFP and *lck* expression in GFP + but not in GFP – sorted Tg(*rag2:GFP*) cells. For all samples (A) & (B) 20,000 cells were sorted directly into the lysis buffer of the RNA isolation (RNAqueous micro or RNeasy plus micro) kit (PDF 874 kb)

Additional file 4: **Figure S3**. Sequencing libraries run in multiple rounds of sequencing introduces substantially larger inter-run variation than the effects imposed by the use of different RNA isolation kit. Heatmap (*left*) and PCA analysis (*right*) of all samples sequenced. A_1 and Q_1 are sequenced on a different sequencing run than A_2a, A_2b, Q_2a, Q_2b, A_unsorted and Q_unsorted (PDF 889 kb)

Additional file 5: Supplementary note 2: protocol for RNA isolation and sequencing of a low number (5000- 200000) of sorted zebrafish cells (PDF 606 kb)

Additional file 6: **Figure S4**. FACS gating strategy used to collect EGFP positive cells from a clutch of Tg(*fli1a:EGFP)* zebrafish. Representative samples of a negative control sample (left) and a Tg(*fli1a*:EGFP) sample (right). This gating strategy was used to first select live cells based on forward and side scatter, with subsequent selection of single cells. Last, GFP positive cells were selected and sorted. No GFP+ cells are seen in control fish (*left*) while 5–15% of the cell population was found to be GFP positive cells in Tg(*fli1a:EGFP)* samples (*right*) (PDF 1027 kb)

## Abbreviations

dpf: days post fertilization
ERE: expressed repetitive elements
FACS: fluorescence-activated cell sorter
GO: Gene Ontology
PCA: Principal component analysis
RQN: RNA quality number
RT-qPCR: reverse transcription quantitative polymerase chain reaction
