# Supplementary material for: Purification of high-quality RNA from a small number of fluorescence activated cell sorted zebrafish cells for RNA sequencing purposes
Source: BMC Genomics. 2019 Mar 20;20:228. doi: 10.1186/s12864-019-5608-2 (PMC6425699; doi:10.1186/s12864-019-5608-2)
Supplement: Supplementary file 1 — Figure S1. Adaptation of the RNA isolation protocol allows purification of small RNA’s (< 200 nt). Fragment analyser electropherogram shown for the RNAqueous micro kit (top) and the RNeasy plus micro kit (bottom) for normal isolation procedure (left) and for RNA isolation with the adapted protocol for small RNA’s as provided in the manual (right). The red arrow indicates presence of small RNA’s in the isolated RNA sample (PDF 1667 kb) [file 12864_2019_5608_MOESM1_ESM.pdf]

Supplemental figure 1

RNAqueous micro kit

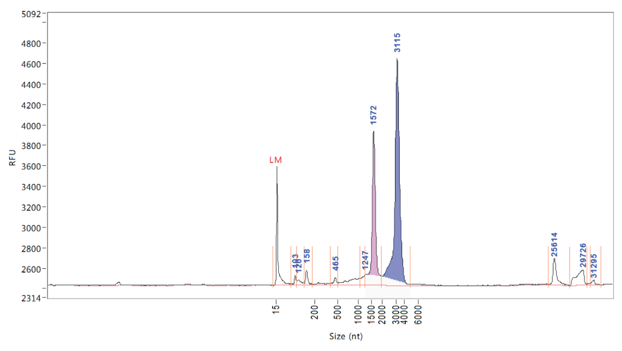

Regular isolation

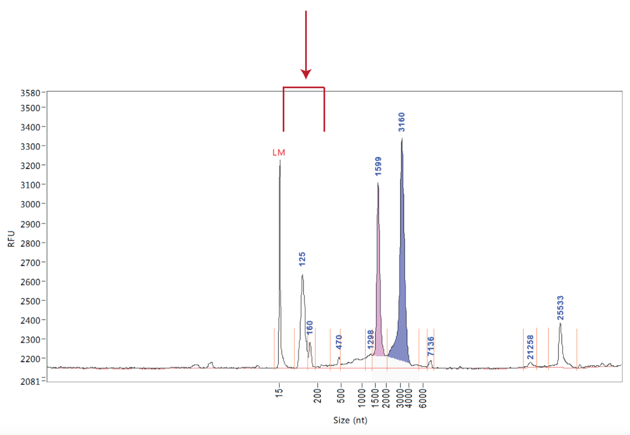

Purification with additional step for small RNA's isolation

RNeasy plus micro kit

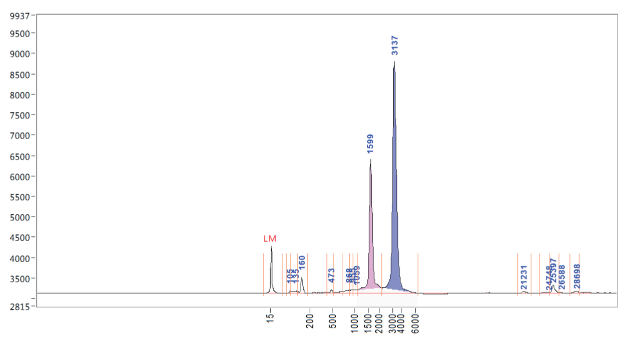

Regular isolation

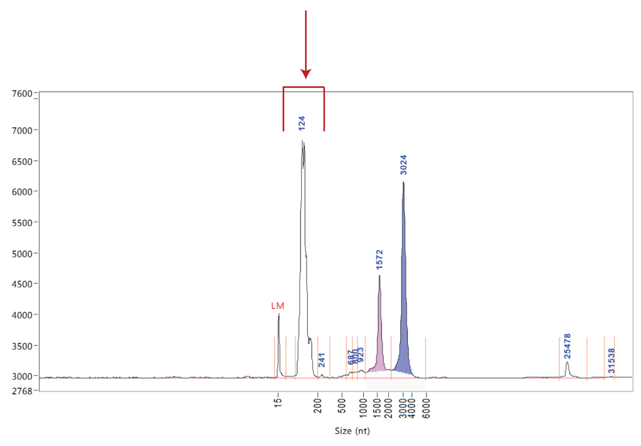

Purification with additional step for small RNA's isolation
