# Supplementary material for: Purification of high-quality RNA from a small number of fluorescence activated cell sorted zebrafish cells for RNA sequencing purposes
Source: BMC Genomics. 2019 Mar 20;20:228. doi: 10.1186/s12864-019-5608-2 (PMC6425699; doi:10.1186/s12864-019-5608-2)
Supplement: Supplementary file 2 — Supplementary note 1: Statistical analysis (DOCX 147 kb) [file 12864_2019_5608_MOESM2_ESM.docx]

**Supplementary note 1: Statistical analysis**

The statistical analyses for detection of gDNA contamination data were performed using the ANOVA variance analysis approach. We analysed if there was a significant difference between the gDNA removal approaches for each RNA isolation kit and each tested reference gene. Two gDNA removal methods were compared pairwise and adjusted p values are given for each comparison.

RNeasy plus micro kit: qPCR assay targeting *elfa*

| **gDNA removal method 1** | **gDNA removal method 2** | **diff** | **lwr** | **upr** | **p adj** |
| --- | --- | --- | --- | --- | --- |
| RNeasy | Heat and Run | 9.210000e-01 | -0.3690408 | 2.211041 | 0.2139727 |
| RNeasy + Heat & Run | Heat & Run | 9.210000e-01 | -0.3690408 | 2.211041 | 0.2139727 |
| No gDNA removal | Heat & Run | -1.345900e+01 | -14.7490408 | -12.168959 | **0.0000000** |
| RNeasy + Heat & Run | RNeasy | -7.105427e-15 | -1.2900408 | 1.290041 | 1.0000000 |
| No gDNA removal | RNeasy | -1.438000e+01 | -15.6700408 | -13.089959 | **0.0000000** |
| No gDNA removal | RNeasy + Heat & Run | -1.438000e+01 | -15.6700408 | -13.089959 | **0.0000000** |

Result: There is a clear significant difference in gDNA contamination without gDNA removal versus one or two gDNA removal steps. Yet, no significant reduction in gDNA contamination is seen when samples are treated with an additional Heat & Run gDNA removal step (Rneasy gDNA removal versus Rneasy plus Heat & Run gDNA removal).

Conclusion: A gDNA removal step is necessary to obtain a pure RNA sample. Yet, an additional gDNA removal step (Heat & Run) does not significantly reduce the gDNA contamination for the RNeasy plus micro kit. This indicates that the gDNA removal step provided by the kit is sufficient.

RNeasy plus micro kit: qPCR assay targeting *loopern4*

| **gDNA removal method 1** | **gDNA removal method 2** | **diff** | **lwr** | **upr** | **p adj** |
| --- | --- | --- | --- | --- | --- |
| RNeasy | Heat and Run | 9.169 | 7.9330527 | 10.404947 | **0.0000000** |
| RNeasy + Heat & Run | Heat & Run | 10.799 | 9.5630527 | 12.034947 | **0.0000000** |
| No gDNA removal | Heat & Run | -6.399 | -7.6349473 | -5.163053 | **0.0000000** |
| RNeasy + Heat & Run | RNeasy | 1.630 | 0.3940527 | 2.865947 | **0.0081291** |
| No gDNA removal | RNeasy | -15.568 | -16.8039473 | -14.332053 | **0.0000000** |
| No gDNA removal | RNeasy + Heat & Run | -17.198 | -18.4339473 | -15.962053 | **0.0000000** |

Result: A qPCR assay targeting *loopern4* to detect gDNA contamination shows there is a significant difference between all the different gDNA elimination approaches. Yet this gDNA contamination is not detectable with a qPCR assay targeting a standard reference gene. *loopern4* is an expressed repeat, so it’s sequence is found in multiple copies in the DNA and therefore expected to be picked up a lot easier by this assay.

Conclusion: This shows that there is only a low amount of gDNA contamination when one or two gDNA elimination steps are performed.

RNAqueous micro kit: qPCR assay targeting *elfa*

| **gDNA removal method 1** | **gDNA removal method 2** | **diff** | **lwr** | **upr** | **p adj** |
| --- | --- | --- | --- | --- | --- |
| RNAqueous | Heat and Run | -4.200 | -9.6975784 | 1.2975784 | 0.1694280 |
| RNAqueous + Heat & Run | Heat & Run | 1.873 | -3.6245784 | 7.3705784 | 0.7654571 |
| No gDNA removal | Heat & Run | -10.262 | -15.7595784 | -4.7644216 | **0.0000000** |
| RNAqueous + Heat & Run | RNAqueous | 6.073 | 0.5754216 | 11.5705784 | **0.0278892** |
| No gDNA removal | RNAqueous | -6.062 | -11.5595784 | -0.5644216 | **0.0282066** |
| No gDNA removal | RNAqueous + Heat & Run | -12.135 | -17.6325784 | -6.6374216 | **0.0000553** |

Result: Here we see a significant difference in gDNA presence without gDNA removal versus one or two gDNA removal steps. In addition, if you add an extra Heat & Run gDNA removal step to the standard procedure (with gDNA elimination provided by the kit), there is a significant decrease of gDNA contamination.

Conclusion: These results indicate that for the RNAqueous micro kit, an additional gDNA elimination step is required. The gDNA removal step provided by the kit is insufficient to clear the RNA from contaminating gDNA.

RNAqueous micro kit: qPCR assay targeting *loopern4*

| **gDNA removal method 1** | **gDNA removal method 2** | **diff** | **lwr** | **upr** | **p adj** |
| --- | --- | --- | --- | --- | --- |
| RNAqueous | Heat and Run | -2.354 | -6.8056347 | 2.0976347 | 0.4531328 |
| RNAqueous + Heat & Run | Heat & Run | 1.875 | -2.5766347 | 6.3266347 | 0.6325820 |
| No gDNA removal | Heat & Run | -6.596 | -11.0476347 | -2.1443653 | **0.0031486** |
| RNAqueous + Heat & Run | RNAqueous | 4.229 | -0.2226347 | 8.6806347 | 0.0656049 |
| No gDNA removal | RNAqueous | -4.242 | -8.6936347 | 0.2096347 | 0.0645827 |
| No gDNA removal | RNAqueous + Heat & Run | -8.471 | -12.9226347 | -4.0193653 | **0.0002854** |

Result: No gDNA removal step versus Heat & Run or versus Heat & Run plus gDNA removal by the kit shows a significant difference in gDNA contamination. There is a large variability in the gDNA removal capacity of the enzyme provided by the kit. Therefore, there can be a high amount of gDNA contamination after the gDNA removal provided by the kit.

Conclusion: The RNAqueous gDNA removal step does not clear the RNA sample from contaminating gDNA. An additional gDNA removal step (Heat & Run) is absolutely necessary to obtain a pure RNA sample.
