# Supplementary figures and images for: Purification of high-quality RNA from a small number of fluorescence activated cell sorted zebrafish cells for RNA sequencing purposes
Source: BMC Genomics. 2019 Mar 20;20:228. doi: 10.1186/s12864-019-5608-2 (PMC6425699; doi:10.1186/s12864-019-5608-2)

Supplemental figure 2

A

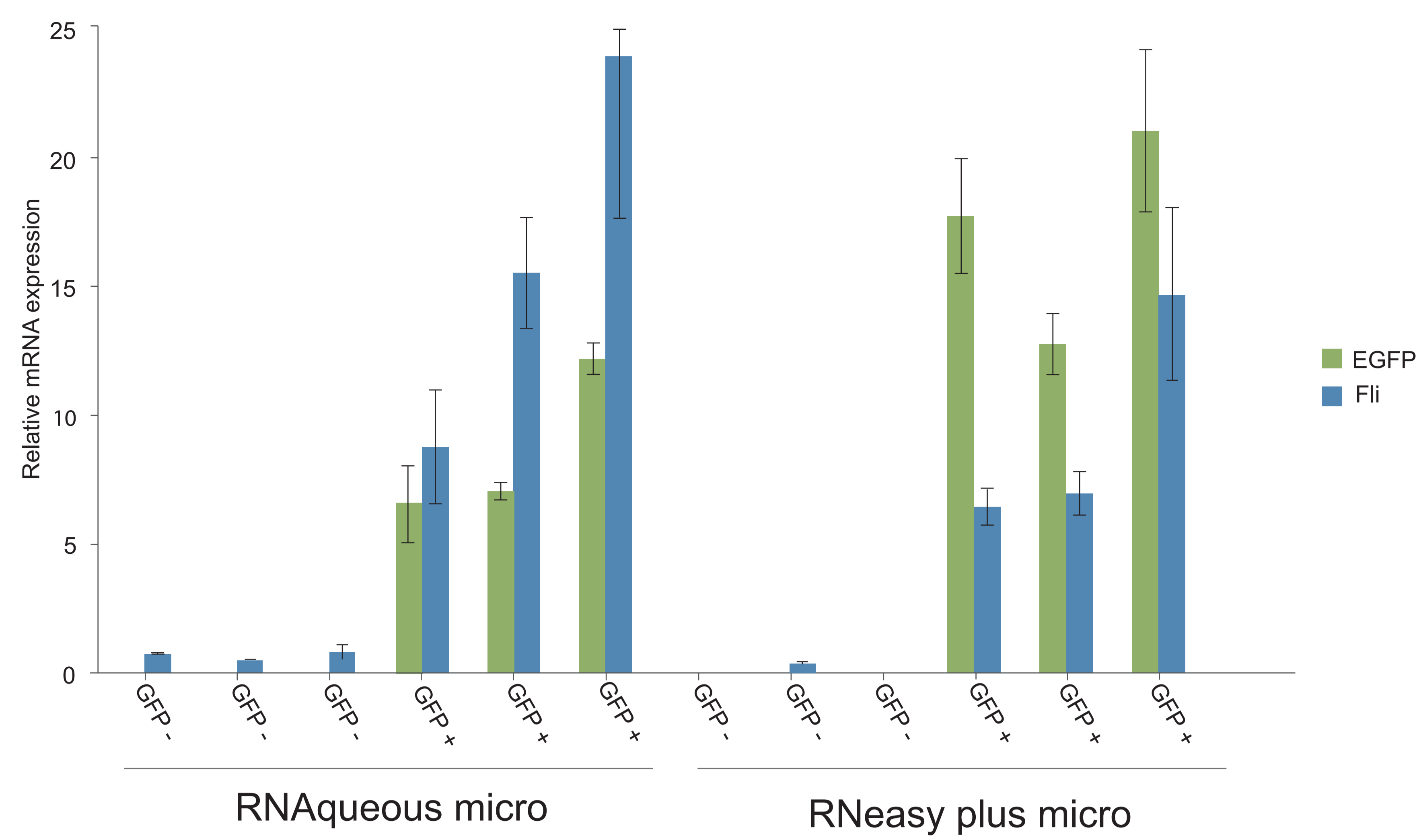

B

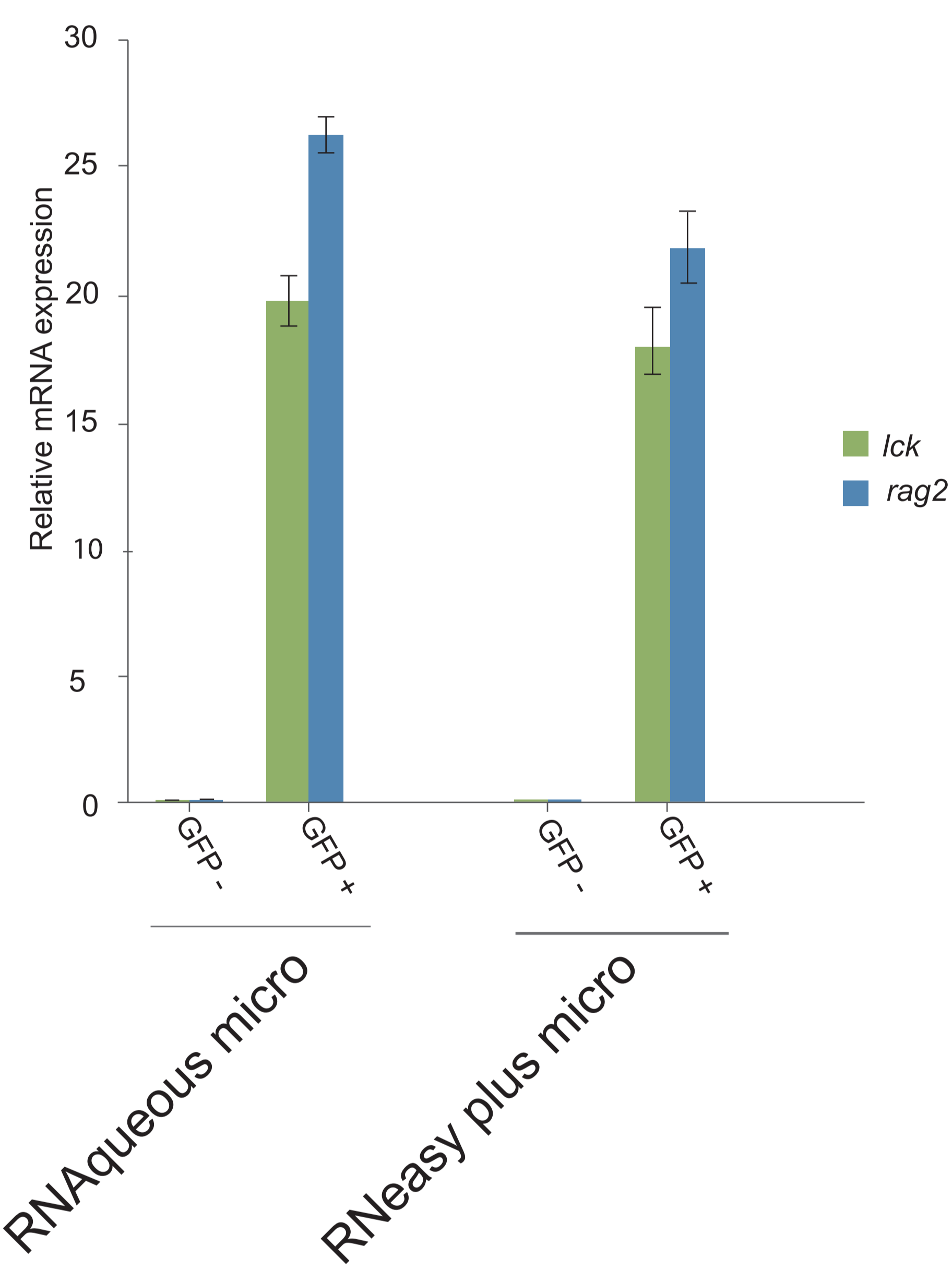

Supplement: Supplementary file 3 — Figure S2. RT-qPCR confirms tissue specific gene expression in tissue specific sorted cells. (A) From a clutch of Tg(fli1a:EGFP) embryos, EGFP positive and EGFP negative cells were sorted. RT-qPCR analysis shows expression of EGFP and fli1a is in the EGFP positive samples but not or negligible in the EGFP negative samples. (B) RT-qPCR analysis confirms GFP and lck expression in GFP + but not in GFP – sorted Tg(rag2:GFP) cells. For all samples (A) & (B) 20,000 cells were sorted directly into the lysis buffer of the RNA isolation (RNAqueous micro or RNeasy plus micro) kit (PDF 874 kb) [file 12864_2019_5608_MOESM3_ESM.pdf]

Supplemental figure 3

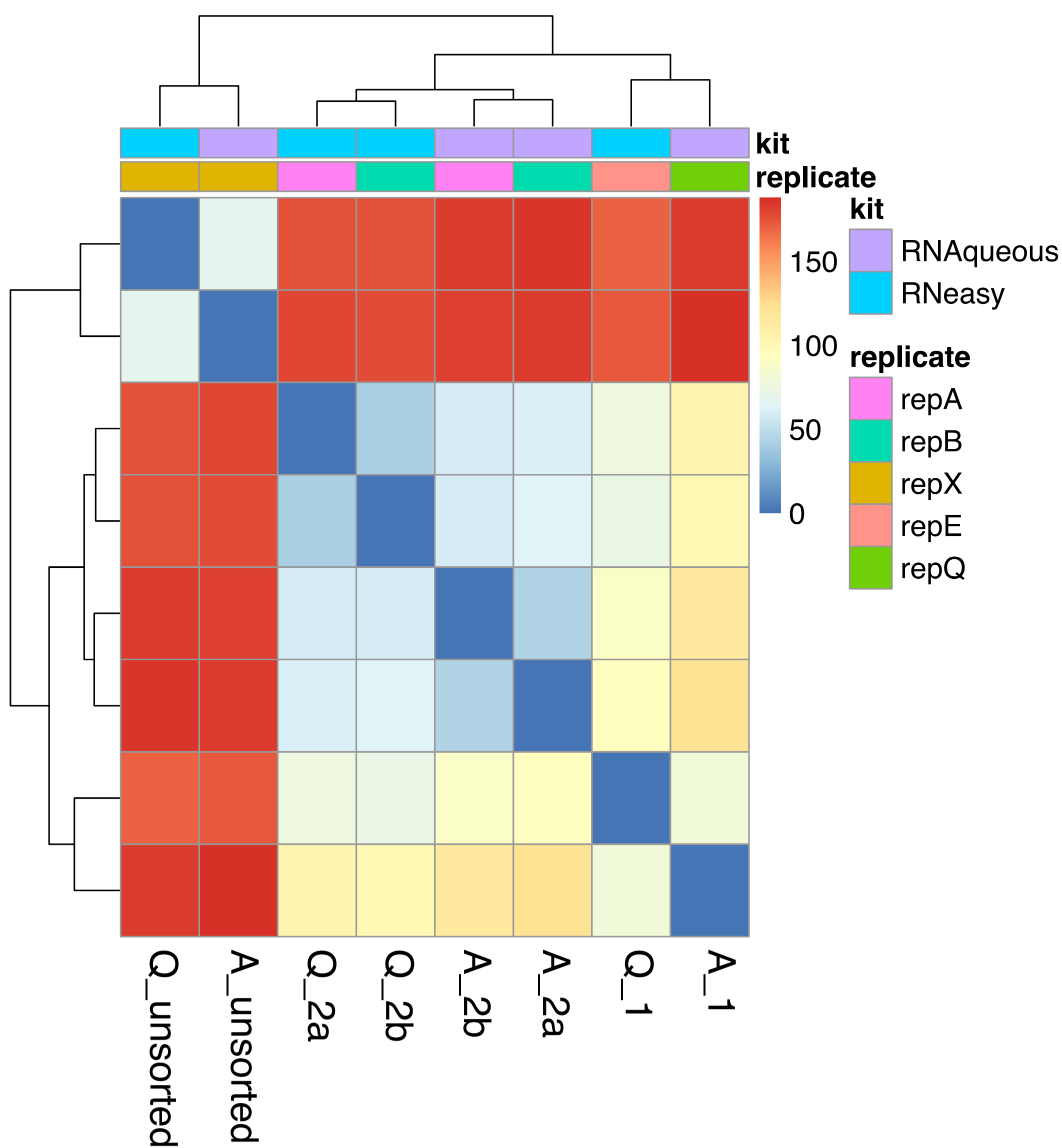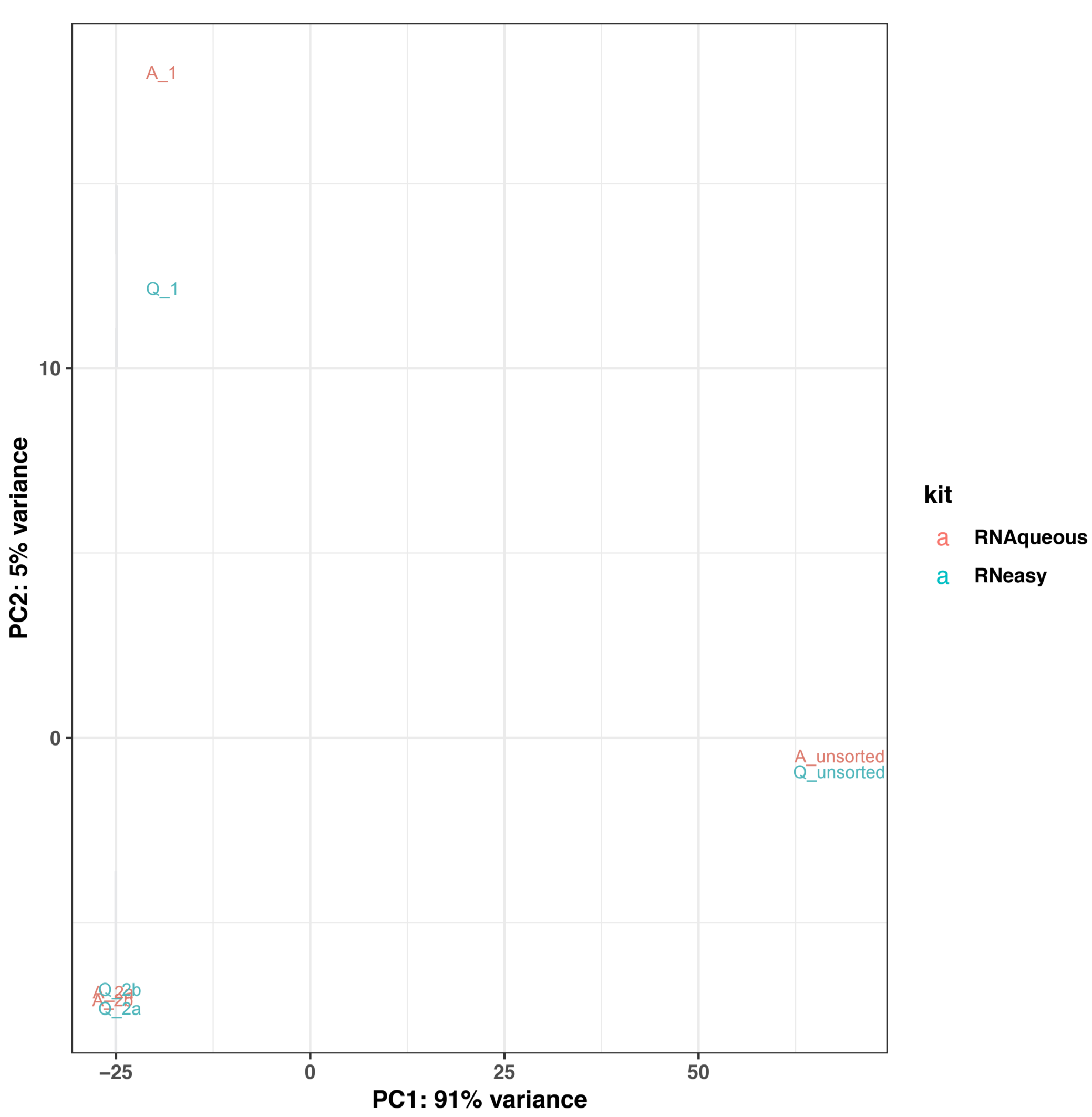

Supplement: Supplementary file 4 — Figure S3. Sequencing libraries run in multiple rounds of sequencing introduces substantially larger inter-run variation than the effects imposed by the use of different RNA isolation kit. Heatmap (left) and PCA analysis (right) of all samples sequenced. A_1 and Q_1 are sequenced on a different sequencing run than A_2a, A_2b, Q_2a, Q_2b, A_unsorted and Q_unsorted (PDF 889 kb) [file 12864_2019_5608_MOESM4_ESM.pdf]

Supplemental figure 4

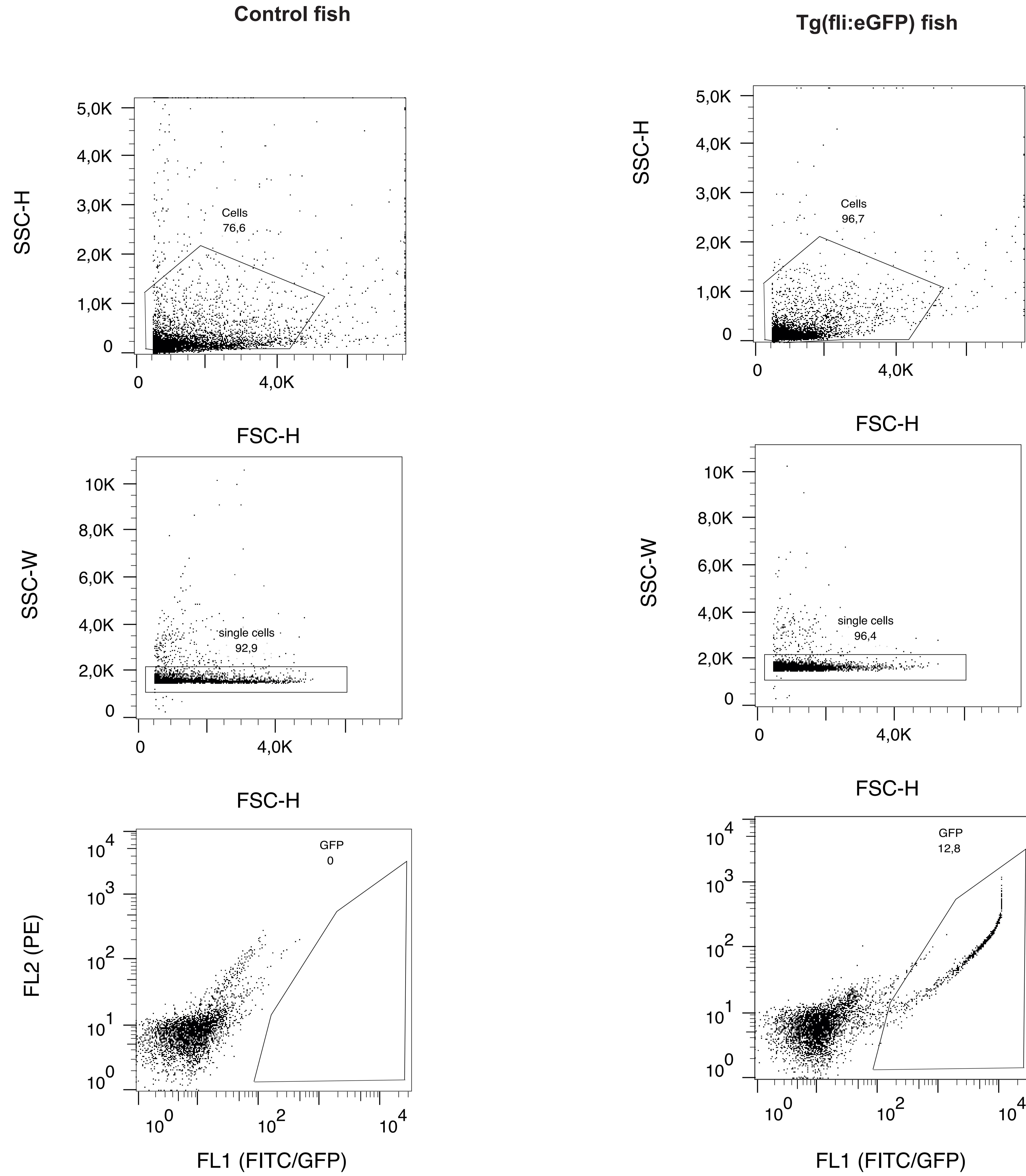

Supplement: Supplementary file 6 — Figure S4. FACS gating strategy used to collect EGFP positive cells from a clutch of Tg(fli1a:EGFP) zebrafish. Representative samples of a negative control sample (left) and a Tg(fli1a:EGFP) sample (right). This gating strategy was used to first select live cells based on forward and side scatter, with subsequent selection of single cells. Last, GFP positive cells were selected and sorted. No GFP+ cells are seen in control fish (left) while 5–15% of the cell population was found to be GFP positive cells in Tg(fli1a:EGFP) samples (right) (PDF 1027 kb) [file 12864_2019_5608_MOESM6_ESM.pdf]
