# Supplementary material for: Purification of high-quality RNA from a small number of fluorescence activated cell sorted zebrafish cells for RNA sequencing purposes
Source: BMC Genomics. 2019 Mar 20;20:228. doi: 10.1186/s12864-019-5608-2 (PMC6425699; doi:10.1186/s12864-019-5608-2)
Supplement: Supplementary file 5 — Supplementary note 2: protocol for RNA isolation and sequencing of a low number (5000- 200000) of sorted zebrafish cells (PDF 606 kb) [file 12864_2019_5608_MOESM5_ESM.pdf]

SUPPLEMENTARY NOTE 2: PROTOCOL FOR RNA ISOLATION AND SEQUENCING OF A LOW NUMBER (5000- 200000) OF SORTED ZEBRAFISH CELLS

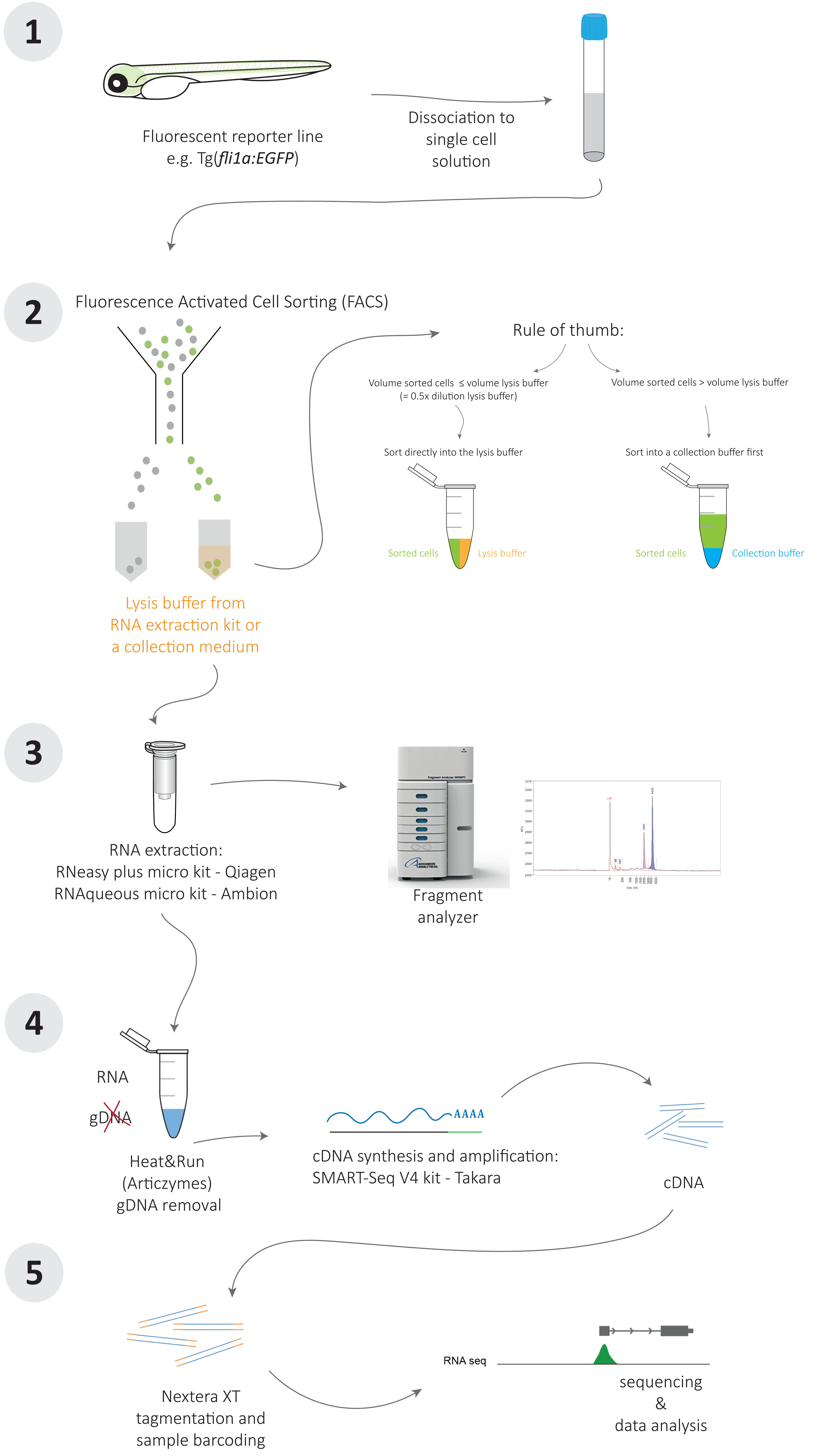

Zebrafish dissociation (*see page 1*)
- embryo & larvae: see step 1A
- adult fish: see step 1B

FAC Sorting of fluorecent cell population
(see page 3)
- Guideliness to choose your collection medium: see step 2

RNA isolation and quality control *(see page 4)*
- Rneasy plus micro kit: see step 3A
- RNAqueous micro kit: see step 3B
- quality control: see step 3C

cDNA synthesis and amplification *(see page 6)*
- additional gDNA removal step: see step 4A
- SMART-seq V4 kit: see step 4B

Library prep and sequencing *(see page 10)*
- Nextera XT: see step 5

1 ZEBRAFISH DISSOCIATION

A) LARVAE

1. Make dissociation solution (0.25% Trypsine, 1 mM EDTA, 1x PBS) and preheat the solution to 28.5 °C prior use.

2. Collect 100–150 larvae and euthanize them (e.g. with an overdose of tricaine). Transfer them into a 35 mm culture dish.

3. Remove all tricaine and add 3 mL dissociation solution.

4. Incubate for roughly 90 min at 28.5 °C in an incubator. During incubation, pipet up and down with a 1 mL tip to dissociate the larvae. Repeat this every 15 min as this will accelerate the dissociation process.

5. Stop the dissociation reaction by adding $CaCl_2$ to a final concentration of 1 mM and fetal calf serum (FCS) to a final concentration of 10%.

6. Transfer cells into a 15 ml tube and centrifuge for 5 min at 800 x g.

7. Rinse the cells in 1 ml of 1x PBS and centrifuge again for 3 min at 800 x g.

8. Resuspend the cells in 1.5 mL of resuspension solution (Leibovitz's L-15 medium + L-Glutamine without Phenol Red, FCS 10%, 0.8 mM $CaCl_2$).

9. Filter the cells 1x through a 70 µm cell strainer, 2x through a 40 µm cell strainer and then transfer the cells into a FACS tube.

10. Put the cells on ice and proceed to FACS sorting immediately.

B) ADULT

1. Euthanize the fish e.g. by an overdose of tricaine.

2. Put the fish in a petri dish. Add 1-2 mL of 0.9 x PBS, 5% FCS. Disaggregate the fish manually by macerating it with the use of a scalpel or a razor blade as much as possible.

3. Cut a piece from the end of a 1000 µl tip to enlarge the tip and pipet the cell suspension up and down to disaggregate the cells even more. Repeat this with a smaller tip opening. Reduce the tip opening stepwise.

4. When applicable, instead of cutting the whole fish, dissect out the tissue/tumor of interest, before disaggregating it manually.

5. Filter the cells 1x through a 70 µm cell strainer, 2x through a 40 µm cell strainer and transfer the cells to a FACS tube.

6. Put the cells on ice and proceed to FACS sorting immediately.

2 FLUORESCENCE ACTIVATED CELL SORTING

The volume of the collected cells determines if it is beneficial for RNA yield and quality to sort directly into the lysis buffer of the RNA isolation kit or to sort into a collection medium. Although sorting directly into the lysis buffer will lyse the cells immediately and protect the RNA, when diluting the lysis buffer too much, it will lose its lysis potential.
Determine the cell volume after sorting your desired cell count and choose the buffer to collect your cells after sorting based on this volume. Following table may serve as a guideline:

	**Cell volume**	**Buffer used to collect sorted cells**
RNeasy plus micro (Qiagen)	< 350	350 µl lysis buffer (buffer RLT + β mercapto-ethanol)
	350 - 700	700 µl lysis buffer (buffer RLT + β mercapto-ethanol)
	> 700	A collection buffer
RNAqueous micro (Ambion)	< 100	100 µl lysis buffer
	100 – 200	200 µl lysis buffer
	>200	A collection buffer

Sorting into the lysis buffer of the RNA isolation kit:

- Add lysis buffer in the tube used to collect the sorted cells.
  Rneasy plus micro kit: 350 µl buffer RLT + 3.5 µl β mercapto-ethanol (or more, see table).
  RNAqueous micro kit: 100 µl lysis buffer (or more, see table).
- Sort the cells.
- Invert the tube a couple times after sorting to make sure that all cells are collected into the lysis buffer.
- Place the sample on ice and proceed with step one of the RNA isolation procedure.
- If desired, the lysate can be stored bij -80 °C until RNA isolation is performed.

Sorting into a collection buffer:

- Used collection buffer: Leibovitz's L-15 medium + L-Glutamine without Phenol Red, FCS 10%, 0.8 mM CaCl2 or 0.9x PBS, 5% FCS.
- Pellet cells after sorting (5 min 800 x g, or 10 min 800 x g when cell volume > 10 ml).
- Discard supernatant.
- Add lysis buffer.
  Rneasy plus micro kit: 350 µl buffer RLT + 3.5 µl β mercapto-ethanol.
  RNAqueous micro kit: 100 µl lysis buffer.
- Proceed to step 1 of the RNA isolation procedure.

3 RNA ISOLATION

See manual of the RNA isolation kit for detailed protocol.

A) RNEASY PLUS MICRO KIT

It is recommended to add β-mercapto ethanol to buffer RLT (lysis buffer) as described in the manual (10 µl β-mercapto ethanol for 1 ml buffer RLT).

For detailed version of the protocol: see manual.

Short version:

1. Transfer the lysate to a gDNA Eliminator spin column placed in a 2 ml collection tube (supplied). Centrifuge 30 s at 10 000 x g.
   Discard the column, and **save the flow-through**.

2. Add 1 volume (usually 350 µl) of 70% ethanol to the flow-through and mix well by pipetting. Do not centrifuge.

3. Transfer the sample, including any precipitate, to a RNeasy MinElute spin column (stored at 4 °C) placed in a 2 ml collection tube. Centrifuge for 15 s at 10 000 x g.
   Discard the flow-through.

4. Add 700 µl Buffer RW1 to the RNeasy MinElute spin column. Centrifuge for 15 s at 10 000 x g. Discard the flow-through.

5. Add 500 µl Buffer RPE to the RNeasy MinElute spin column. Centrifuge for 15 s at 10 000 x g. Discard the flow-through.

6. Add 500 µl of 80% ethanol to the RNeasy MinElute spin column. Centrifuge for 2 min at 10 000 x g to wash the spin colomn membrane.
   Discard the collection tube with the flow-through.

7. Place the RNeasy MinElute spin column in a new 2 ml collection tube. Open the lid of the spin colomn and centrifuge at full speed for 5 min to dry the membrane.
   Discard the collection tube with the flow-through.

8. Place the RNeasy MinElute spin column in a new 1.5 ml tube (not supplied).
   Add 14 µl RNase-free water directly to the center of the spin column membrane.
   Close the lid gently and centrifuge for 1 min at full speed to elute the RNA.

9. Store at -80 °C

B) RNA ISOLATION: RNAQUEOUS MICRO KIT

For detailed version of the protocol: see manual.

Short version:

I. Preparation of materials

1. Warm Wash Solution 2/3 to room temperature.
2. Heat an aliquot of 'Elution Solution' in a heat block set to 75 °C ➔± 50 µL of Elution Solution per sample

II. RNA isolation procedure

3. For a standard prep of 100 µl of lysate, add 50 µl of 100% ethanol, and vortex briefly but thoroughly.
4. Load the lysate/ethanol mixture (up to 150 µl) onto a Micro Filter Cartridge Assembly.
5. Centrifuge for 10 s at maximum speed to pass the solution through the filter.
6. Add 180 µl of Wash Solution 1 to the filter.
7. Centrifuge for ~10 s to pass the solution through the filter.
8. Add 180 µl of Wash Solution 2/3 (working solution mixed with ethanol) to the filter.
9. Centrifuge for ~10 s to pass the solution through the filter.
10. Repeat with a second 180 µl aliquot of Wash Solution 2/3.
11. Pour out the flow-through.
12. Replace the Micro Filter Cartridge into the same Collection Tube, and centrifuge at maximum speed for 1 min to remove residual fluid and dry the filter.
13. Transfer the Micro Filter Cartridge into a new 1.5 ml RNase-free tube.
14. Apply 10 µl of Elution Solution, preheated to 75 °C, to the center of the filter.
15. Store the assembly for 1 min at room temperature.
16. Centrifuge the assembly for ~30 s to elute the RNA from the filter.
17. Repeat with a second 10 µl aliquot of preheated Elution Solution, collecting the eluate in the same Micro Elution Tube.

III. DNase I treatment and DNase inactivation

18. Set temperature of the heatblock at 37 °C.
19. Add 2 µl 10x DNase I Buffer (1/10 volume) and 1 µl of DNase I to the sample and mix gently but thoroughly.
20. Incubate the DNase reaction 20 min at 37 °C.
21. Remove the DNase Inactivation Reagent from –20 °C and allow it to thaw at room temperature during this incubation.
22. Vortex the DNase Inactivation Reagent vigorously to completely resuspend the slurry.
23. Add 2.3 µl DNase Inactivation Reagent.
24. Store the reaction at room temperature for 2 min, vortexing once during this interval to disperse the DNase Inactivation Reagent.
25. Centrifuge 1.5 min at maximum speed to pellet the DNase Inactivation Reagent.
26. Transfer the RNA to a fresh RNase-free tube and store at -80 °C.

C) RNA QUALITY CHECK

Determine the quality and the yield of the extracted RNA with the Fragment analyzer or a similar method. With this method, RQN values >7 can be obtained.

4 CDNA SYNTHESIS AND AMPLIFICATION

A) ADDITIONAL gDNA REMOVAL STEP: HEAT&RUN

Prior to cDNA synthesis, an additional gDNA removal step is performed to completely remove contaminating gDNA.

- Mix sample with Heat&Run (ArticZymes) kit:
    - 8 - 50 µl RNA
    - 1/10th of RNA volume 10x Rxn buffer
    - 1 µl HL- dsDNase

- Incubate:
    10 min 37 °C
    5 min 58 °C

- Proceed immediately to cDNA synthesis as no additional purification is needed.

B) SMART-SEQ V4 ULTRA LOW INPUT RNA KIT FOR SEQUENCING

I. First-Strand cDNA synthesis

1. Thaw the 5x Ultra Low First-Strand Buffer (red) **at room temperature**, the 10x lysis Buffer (no color) and RNase Inhibitor (white) **on ice**. Vortex each reagent and spin down briefly.
2. Prepare a stock solution of 10x Reaction Buffer as indicated (1 µl for each sample):

    9.5 µl 10x lysis Buffer (no color)
    0.5 µl RNase inhibitor (white)
    10 µl total volume

3. Mix briefly and spin down *(contains detergent => avoid bubbles when mixing!)*
4. First dilute the positive control: use same input as test samples.

    When diluting, at 1 µl RNase inhibitor per 50 µl.
    E.g. (1/100): 97 µl H_20, 2 µl RNase inhibitor and 1 µl control.

5. Prepare each sample in individual 0.2 ml RNase-free PCR tubes:

	**POSITIVE CONTROL**	**TEST SAMPLE**
**10X REACTION BUFFER**	1 µl	1 µl
**DILUTED CONTROL RNA**	0.5 µl	/
**SAMPLE RNA**	/	1-9.5 µl
**NUCLEASE FREE WATER**	9 µl	up to 8.5µl
**TOTAL VOLUME**	**10.5 µl**	**10.5 µl**

6. Place the samples on ice and add 2 µl of the 3' SMART-Seq CDS Primer IIA (blue) (total volume = 12.5 µl). Mix well by gently vortexing and then spin the tubes.
7. Incubate the tubes at 72 °C in a preheated, hot-lid thermal cycler for 3 min.
8. Prepare the Master Mix for all the samples (10% extra):

    4 µl 5x Ultra Low First-Strand Buffer (red)

1 µl SMART-Seq v4 Oligonucleotide (pink)
0.5 µl RNase Inhibitor (white)

5.5 µl for each sample

9. After the incubation (3 min 72 °C), place the samples on ice for 2 min.
10. Preheat the thermal cycler to 42 °C.
11. Add 2 µl per reaction (plus 10%), of the SMARTScribe Reverse Transcriptase (purple) to the MasterMix (just prior to use). Mix the MasterMix well by vortexing and spin down.
12. Add 7.5 µl of MasterMix to each reaction tube. Mix by gently pipetting and spin down.
13. Place the tubes in a thermal cycler with a heated lid, preheated to 42 °C. Run the following program: 42 °C 90 min
    70 °C 10 min
    4 °C forever

PAUSE POINT: The tubes can be stored overnight at 4 °C.

II. cDNA amplification by LD PCR

1. Thaw all reagents needed for PCR on ice. Gently vortex each reagent and spin down.
2. Prepare enough PCR MasterMix for all reactions (plus 10%).
   25 µl 2x SeqAmp PCR Buffer (no color)
   1 µl PCR Primer II A (green)
   1 µl SeqAmp DNA Polymerase (no color)
   3 µl Nuclease -free water
   30 µl Total volume per reaction
3. Mix the MasterMix by vortexing and spin down.
4. Add 30 µl of PCR MasterMix to each tube containing 20 µl of first-strand cDNA product. Mix well and briefly spin down.
5. Place the tubes in a preheated thermal cycler and run following program:

	1 min	95 °C
x cycli*	10 s	98 °C
	30 s	65 °C
	10 min	68 °C
	10 min	72 °C
	1 min	4 °C

*Number of cycli depends on amount of total RNA used for the first-strand cDNA synthesis:

for 10 ng 7-8 cycli
for 1 ng 10-11 cycli
for 100 pg 14-15 cycli
for 10 pg 17-18 cycli

PAUSE POINT: The tubes can be stored overnight at 4 °C.

III. Purification of Amplified cDNA using the Agencourt AMPure XP Kit

1. Bring bead aliquots to room temperature at least 30 min before use.

2. Add 1 µl of 10x lysis buffer (no color) to each PCR product.
3. Vortex AMPure xP beads and add 50 µl to each sample. Mix by vortexing or pipetting up and down at least 10 times to mix thoroughly.
4. Incubate at room temperature for 8 min to let the cDNA bind to the beads.
5. Spin the samples and place them on the magnetic separation device for 5 min or until the liquid appears completely clear and there are no beads left in the supernatant.
6. While samples are on the separation device, add 200 µl freshly made 80% ethanol to each sample without disturbing the beads.
7. Wait for 30 s and carefully pipette the supernatant containing contaminants and discard; cDNA will remain bound to the beads during the washing process.
8. Repeat the ethanol wash step once (6 and 7)
9. Briefly spin the samples and bring them back on the separation device for 30 s and remove the remaining ethanol.
10. Place the samples at room temperature for 2-2.5 min until the pellet is no longer shiny, but before a crack appears.
11. Once the beads are dry, add 17 µl of elution buffer to cover the bead pellet.
12. Remove the samples from the magnetic separation device and mix to resuspend the beads.
13. Incubate at room temperature for 2 min to rehydrate.
14. Briefly spin the samples and place them back on the magnetic separation device for 1 min or longer, until the solution is completely clear.
15. Transfer the clear supernatant containing purified cDNA from each well to a nuclease-free, low adhesion tube. Store at -20 °C.

STOPPING POINT: The tubes may be stored at -20 °C indefinitely.

IV. Validation using the Agilent 2100 Bioanalyser

1. Aliquot 1 µl of the amplified cDNA for validation using the Agilent 2100 Bioanalyser and Agilent's High Sensitivity DNA kit.
2. See user manual for instructions.
3. Compare results for your samples and controls to verify whether the sample is suitable for further processing. Successful cDNA synthesis and amplification should yield a distinct peak spanning 400 bp to 10 000 bp, peaked at 2500 bp for the positive control, yielding approximately 3.4-17 ng of cDNA.

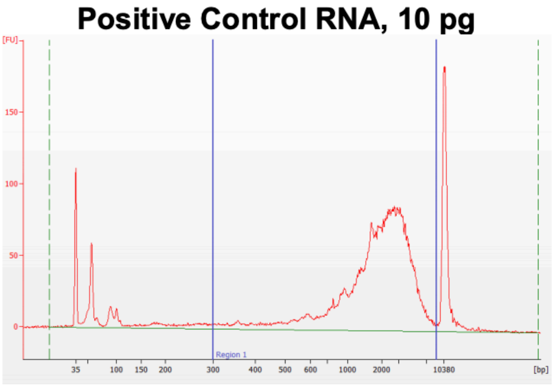
Positive Control RNA, 10 pg
[FU]
150
100
50
0
Region 1
35
100
150
200
300
400
500
600
1000
2000
10380
[bp]

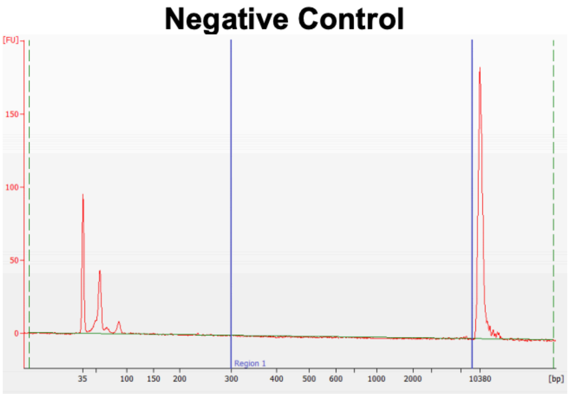
Negative Control
[FU]
150
100
50
0
Region 1
35
100
150
200
300
400
500
600
1000
2000
10380
[bp]

5 NEXTERA XT LIBRARY PREP (ILLUMINA)

See manual for detailed protocol.

Short version:

I. Tagment Genomic DNA

1. Measure cDNA with Qubit high sensitivity kit.
2. Dilute cDNA to 0.2 ng/µl.
3. Add following volumes in PCR tube:
   - 10 µl TD
   - 5 µl diluted cDNA (= 1 ng)
   - 5 µl ATM
4. Pipet to mix.
5. Centrifuge 280 x g, 1 min.
6. Place samples in thermal cycler (choose preheat lit option), **immediately proceed to step 7 when sample reaches 10 °C**:
   - 55 °C, 5 min
   - hold at 10 °C
7. Add 5µl NT, pipette to mix.
8. Centrifuge at 280 x g at 20 °C.
9. Incubate at room temperature for 5 min.

II. Amplify Libraries

1. Thaw indexes at room temperature for 20 min.
2. Thaw NPM on ice for 20 min.
3. Add 5 µl of index 1 (i7) adaptor (Nxxx).
4. Add 5 µl of index 2 (i5) adaptor (Sxxx).
   ! make sure samples have different index combinations!
5. Add 15 µl NPM.
6. Centrifuge 280 x g 1 min.
7. Place in thermal cycler (choose preheat option)
   - 72 °C 3 min
   - 95 °C 30 s
   - 12 cycles of:
     - 95 °C 10 s
     - 55 °C 30 s
     - 72 °C 30 s
   - 72 °C 5 min
   - hold 10 °C

III. Clean up libraries

1. Let AMPure XP beads stand on the benchtop for 30 min to bring to room temperature.
2. Thaw RSB at roomtemperature.
3. Prepare fresh 80% EtOH.
4. Centrifuge 280 x g 1 min.
5. Add 30 µl AMPure XP beads.

6. Shake at 1800 RPM for 2 min.
7. Incubate at room temperature for 5 min.
8. Place on magnetic stand and wait until the liquid is clear (+/- 2 min).
9. Remove and discard supernatant.
10. Wash 2 times as follows:
    - add 200 µl fresh 80% EtOH
    - incubate on magnetic stand
    - remove supernatant
11. Remove residual 80% EtOH.
12. Air dry on magnetic stand for 15 min.
13. Remove from magnetic stand.
14. Add 52,5 µl RSB to each well.
15. Shake at 1800 rmp for 2 min.
16. Place on magnetic stand and wait until liquid is clear.
17. Transfer 50 µl supernatant to new tube.

IV. Check libraries

1. Run 1 µl undiluted library on Agilent Technology 2100 bioanalyzer using a high sensitivity DNA chip.
2. Expected fragments: borad size dirstibution of 250 – 1500 bp.

V. Quantify libraries and pool equimolar

Use Kapa library quantification kit from Illumina.
